# Two separate, large cohorts reveal potential modifiers of age-associated variation in visual reaction time performance

**DOI:** 10.1038/s41514-021-00067-6

**Published:** 2021-07-01

**Authors:** J. S. Talboom, M. D. De Both, M. A. Naymik, A. M. Schmidt, C. R. Lewis, W. M. Jepsen, A. K. Håberg, T. Rundek, B. E. Levin, S. Hoscheidt, Y. Bolla, R. D. Brinton, N. J. Schork, M. Hay, C. A. Barnes, E. Glisky, L. Ryan, M. J. Huentelman

**Affiliations:** 1 The Translational Genomics Research Institute (TGen), Phoenix, AZ USA; 2 Arizona Alzheimer’s Consortium, Phoenix, AZ USA; 3 Norwegian University of Science and Technology, Trondheim, Norway; 4 University of Miami Miller School of Medicine and Evelyn F. McKnight Brain Institute, Miami, FL USA; 5 University of Arizona, Tucson, AZ USA; 6 City of Hope National Medical Center, Duarte, CA USA

**Keywords:** Alzheimer's disease, Risk factors

## Abstract

To identify potential factors influencing age-related cognitive decline and disease, we created MindCrowd. MindCrowd is a cross-sectional web-based assessment of simple visual (sv) reaction time (RT) and paired-associate learning (PAL). svRT and PAL results were combined with 22 survey questions. Analysis of svRT revealed education and stroke as potential modifiers of changes in processing speed and memory from younger to older ages (*n*_total_ = 75,666, *n*_women_ = 47,700, *n*_men_ = 27,966; ages 18–85 years old, mean (*M*)_Age_ = 46.54, standard deviation *(SD)*_*Age*_ = 18.40). To complement this work, we evaluated complex visual recognition reaction time (cvrRT) in the UK Biobank (*n*_total_ = 158,249 *n*_women_ = 89,333 *n*_men_ = 68,916; ages 40–70 years old, *M*_Age_ = 55.81, SD_Age_ = 7.72). Similarities between the UK Biobank and MindCrowd were assessed using a subset of MindCrowd (UKBb MindCrowd) selected to mirror the UK Biobank demographics (*n*_total_ = 39,795, *n*_women_ = 29,640, *n*_men_ = 10,155; ages 40–70 years old, *M*_Age_ = 56.59, SD_Age_ = 8.16). An identical linear model (LM) was used to assess both cohorts. Analyses revealed similarities between MindCrowd and the UK Biobank across most results. Divergent findings from the UK Biobank included (1) a first-degree family history of Alzheimer’s disease (FHAD) was associated with longer cvrRT. (2) Men with the least education were associated with longer cvrRTs comparable to women across all educational attainment levels. Divergent findings from UKBb MindCrowd included more education being associated with shorter svRTs and a history of smoking with longer svRTs from younger to older ages.

## Introduction

Reaction time (RT), an index of processing speed or efficiency in the central nervous system (CNS)^1^, is an essential factor in higher cognitive function^2,3^ and is profoundly affected by age^4^. In fact, of the studied demographics, age is the main factor known to influence RT^4^. Processing speed is an important limiting factor for most aspects of cognition during aging, most notably memory^5,6^. In studies where processing speed was used as a covariate, the age-related variance in various episodic memory measures was reduced or even eliminated^7,8^. Moreover, studies comparing varied factors and tests of age-related episodic memory deficit implicate age-related decline in processing speed as the main mediator^9–11^. These findings collectively suggest that RT is a useful index of age-related cognitive decline, healthy brain aging, and neurodevelopment.

RT can be operationally defined as “simple,” which typically involves a non-choice reaction to a visual stimulus (svRT). RT can also be operationally defined as “complex,” which involves a reaction to one or more visual stimuli after recognition (cvrRT) of correct stimuli and inhibiting incorrect stimuli^12^. svRT demonstrates variability between individuals, which is akin to paired-associate learning (PAL) and is influenced by genetic and environmental factors^13^. In addition, svRT effects are well noted across the field of neurology; for example, AD and stroke patients show lengthened svRT and higher inter-individual variability^14,15^. However, due to the limitations of traditional research methods, the body of work concerning RT examined only limited ranges of demographic, health, medical, and lifestyle factors in small cohorts. For example, prior work’s demographics consisted of college-aged students, well-educated older adults^16–18^, or athletes^19–21^.

Further, with notable exceptions^22–24^, many studies had few participants (e.g., *n* < 1000) and were therefore powered to detect only variables with large effect size and to lead to spurious non-replicable findings^25^. Consequently, many RT studies had minimal ability to reveal low-frequency factors or those with subtle effect sizes and conduct more sophisticated analyses (e.g., ANOVA vs. Growth Modeling) to find interactions and moderators. Collectively, this suggests that if RT performance can inform models of disease or normative and atypical aging, we need a deeper understanding of the normal variation of RT and the genetic and environmental factors associated with RT performance.

This study aimed to characterize RT across a broad range of demographic, health, medical, and lifestyle variables commonly associated with cognitive performance and AD risk. To do this, we utilized both the MindCrowd and UK Biobank cohorts^26,27^, comprising over 233 thousand combined participants, to model RT as a function of 11 or more demographic, health, medical, and lifestyle factors. These factors have been previously associated with aging and cognition^28–33^. Based on our prior work and earlier RT research^34^, we hypothesized that RT, via its structure of factor association and modifications, would reveal meaningful connections to healthy brain aging.

## Results

### MindCrowd

As of March 13th, 2020, after filtering (see Data Quality Control in “Methods” section), MindCrowd, had recruited 75,666 qualified participants (see Table 1 for Sociodemographic Characteristics and Supplementary Fig. 1A for a histogram of age). We modeled svRT as a function of Age^3^ and PAL Performance^3^ (i.e., curvilinear associations), as well as 20 other factors (see Supplementary Fig. 2 for diagnostic regression plots, and Table 2 for each analysis’ *n*). The omnibus model was significant (*F*_omnibus_[58, 73406] = 858.20, *p*_omnibus_ < 2.2e−16, Adjusted *R*^2^ = 0.40).

**Table 1:** MindCrowd, UKBb MindCrowd, and UK Biobank’s sociodemographic characteristics.

Cohort	Descriptive or factor level	*n*	%
1. MindCrowd 18–85 years: age	M = 46.54 SD = 18.40	75,666	100
UKBb MindCrowd 40–70 years: age	M = 56.59 SD = 8.16	39,795	100
UK Biobank 40–70 years: age	M = 55.81 SD = 7.72	158,249	100
2. MindCrowd 18–85 years: biological sex	Women	47,700	63.08
	Men	27,966	36.91
UKBb MindCrowd 40–70 years: biological sex	Women	29,640	74.51
	Men	10,155	25.49
UK Biobank 40–70 years: biological sex	Women	89,333	56.45
	Men	68,916	43.55
3. MindCrowd 18–85 years: Race	Asian	3511	4.64
	Black/African American	1570	2.07
	Mixed	497	0.66
	Native American	447	0.59
	Native Hawaiian/Pacific Islander	271	0.36
	White	68,450	90.46
UKBb MindCrowd 40–70 years: Race	Asian	750	1.88
	Black/African American	740	1.86
	Mixed	185	0.46
	White	37,446	94.10
UK Biobank 40–70 years: Race	Asian	1612	1.02
	Black/African American	980	0.62
	Mixed	847	0.54
	White	154,810	97.83
4. MindCrowd 18–85 years: FHAD	True	17,847	23.59
	False	57,819	76.41
UKBB MindCrowd 40–70 years: FHAD	True	13,748	34.59
	False	26,047	65.41
UK Biobank 40–70 years: FHAD	True	19,742	12.48
	False	138,507	87.52
5. MindCrowd 18–85 years: handedness	Left	8449	11.17
	Right	66,903	88.42
UKBb MindCrowd 40–70 years: handedness	Left	4520	11.36
	Right	35,034	88.04
UK Biobank 40–70 years: handedness	Left	15,287	9.66
	Right	142,962	90.34
6. MindCrowd 18–85 years:	No high school diploma	1881	2.49
Educational attainment	High school diploma	6695	8.85
	Some college	22,950	30.33
	College degree	44,140	58.34
UKBb MindCrowd 40–70 years:	No high school diploma	605	1.52
Educational attainment	High school diploma	3176	7.98
	Some college	11,139	27.99
	College degree	24,875	62.51
UK Biobank 40–70 years:	No high school diploma	10,978	6.94
Educational attainment	High school diploma	46,248	29.23
	Some college	77,271	48.83
	College degree	23,752	15.01

List of ns and related percentages of commonly reported sociodemographic factors from MindCrowd, UKBb MindCrowd, and the UK Biobank.

**Table 2:** Summary of MindCrowd’s sample sizes (*n*).

Factor	*n*
Biological sex	Women = 47,700
	Men = 27,966
Educational attainment	No high school diploma = 1881
	High school diploma = 6695
	Some college = 22,950
	College degree = 44,140
Handedness	Left-handed = 8449
	Right-handed = 66,903
Daily medications taken	None = 33,672
	One = 14,409
	Two = 9651
	Three = 6656
	Four = 10,769
Reported dizziness	Dizziness reported = 4749
	No dizziness reported = 70,917
Smoking status	Smoker = 5793
	Non-smoker = 69,873
Diabetes mellitus	Diabetes reported = 3887
	No diabetes reported = 71,779
Reported stroke	Stroke reported = 765
	No stroke reported = 74,901

*n* listed for each of MindCrowd’s multiple regression coefficients (i.e., linear model [LM] factors).

### MindCrowd Curvilinear associations: age and paired-associate learning (PAL)

**Fig. 1:**
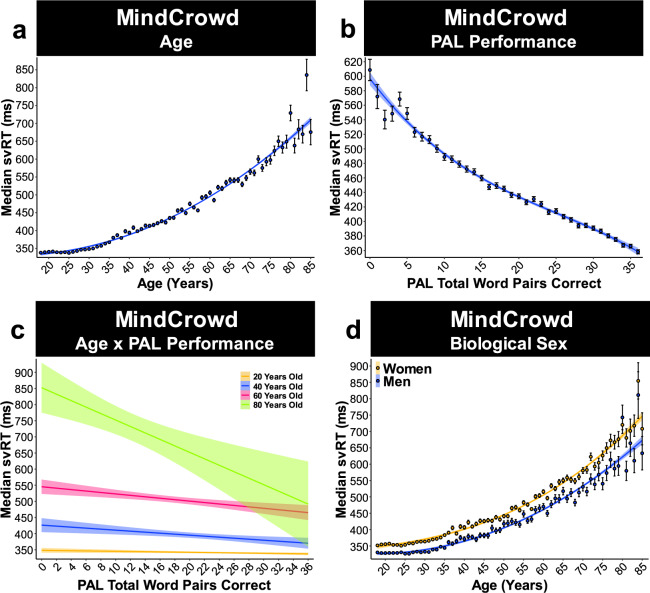
MindCrowd: age, paired-associate learning (PAL), and biological sex. MindCrowd analysis (ages 18–85) of simple visual reaction time (svRT). **a** Linear model fits (line fill ±95% CI, error bars ± SEM) of the median svRT by Age3 (curvilinear model). There was a curvilinear relationship between svRT and Age^1^ (*β*_Age_1 = 7.07, *p*_Age_1 = 3.06E − 18), Age^2^ (*β*_Age2_ = −0.15, *p*_Age2_ = 3.23E − 17), and Age^3^ (*β*_Age3_ = 1.47E − 03, *p*_Age3_ < 1.46E − 34, *n* = 75,666). **b** Linear model fits (line fill ±95% CI, error bars ± SEM) of the median svRT by Age3 (curvilinear model). There was a curvilinear relationship between svRT and paired-associate learning (PAL) performance PAL Performance^1^ (*β*_PAL1_ = −8.89, *p*_PAL1_ = 3.77E − 34), PAL Performance^2^ (*β*_PAL2_ = 0.32, *p*_PALl2_ = 2.35E − 14), and PAL Performance^3^ (*β*_PAL3_ = −4.13E − 03, *p*_PAL3_ = 7.28E − 09, *n* = 75,666). **c** Simple slope analysis of the linear model fit (line fill ±95% CI, error bars ± SEM) for the Median svRT × PAL Performance interaction (*β*_Age*PAL_ = −0.07, *p*_Age*PAL_ = 1.26E − 59). At 20 (*β*_Age20*PAL_ = −5.48, *p*_Age20*PAL_ < 0.00, *n* = 1985), 40 (*β*_Age40*PAL_ = −6.41, *p*_Age40*PAL_ < 0.00, *n* = 739), 60 (*β*_Age60*PAL_ = −7.75, *p*_Age60*PAL_ < 0.00, *n* = 1789), and 80 (*β*_Age80*PAL_ = −9.08, *p*_Age80*PAL_ < 0.00, *n* = 344) years of age. **d** Linear model fits (line fill ±95% CI, error bars ± SEM) of the median svRT by Age^3^ (curvilinear model) with lines split by Biological Sex. Being a woman was associated with longer svRT compared to being a man from younger to older ages (*β*_Sex_ = −34.26, *p*_Sex_ = 1.26E − 163, *n*_Women_ = 47,700, *n*_Men_ = 27,966).

Our model revealed that all three Age polynomials were significantly associated with svRT. Age^1^ (i.e., linear association, first-degree polynomial, aka slope), Age^2^ (i.e., quadratic association, second-degree polynomial), and Age^3^ (i.e., cubic association, third-degree polynomial). On average from younger to older Age, a one-year difference (*X* = 1). (1) Age^1^ (shift in Y; *p*_Age1_ = 3.06E − 18) was associated with 7 ms longer svRT. (2) Age^2^ (shift in Age^1^; *p*_Age2_ = 3.23E − 17) was associated with 0.15 ms of added svRT length (i.e., 7 + .15 ms/year, Fig. 1a). (3) Age^3^ (shift in Age^2^; *p*_Age3_ = 1.46E − 34) was associated with a negligible 1.47E − 03 ms shift in added svRT length (i.e., 7 + (0.15 + 1.47E − 03) ms/year, Fig. 1a). In contrast to Age’s association with longer svRT, each word pair correct for PAL Performance. (1) PAL^1^ (*p*_PAL1_ = 3.77E − 34) was associated 9 ms shorter svRT. (2) PAL^2^ (*p*_PAL2_ = 2.35E − 14) was associated with 0.32 ms of additional svRT shortening (i.e., 9 + 0.32 ms/year, Fig. 1b). (3) PAL^3^ (*p*_PAL3_ = 7.28E−09) was associated with a small 4.13E − 04 ms shift in added svRT shortening (i.e., 9 + (0.32 + 4.13E − 04) ms/year Fig. 1b).

### MindCrowd: sex, education, and handedness

**Fig. 2:**
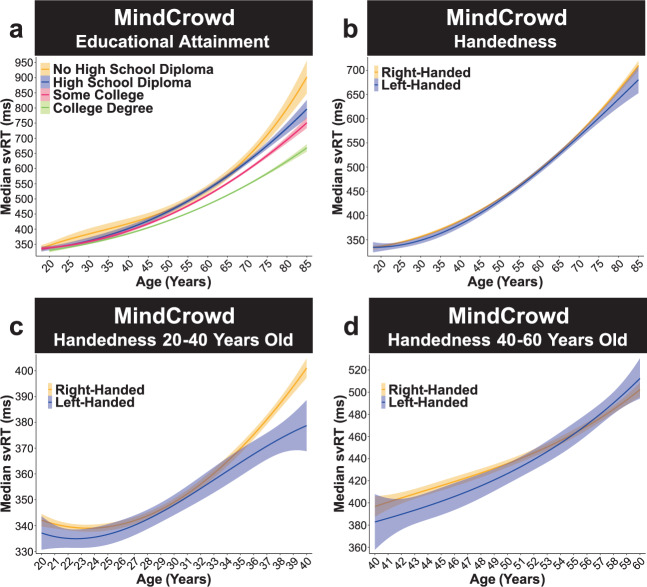
MindCrowd: educational attainment and handedness. MindCrowd analysis (ages 18–85) of simple visual reaction time (svRT). **a** Linear model fits (line fill ±95% CI) of the median svRT by Age^3^ (curvilinear model) with lines split by Educational Attainment. Participants who had “Some College” (*β*_College_ = −14.73, *p*_College_ = 8.74E − 05, *n* = 22,950), or a “College Degree” (*β*_CDegree_ = −31.78, *p*_CDegree_ = 2.95E − 17, *n* = 44,140) were shorter than those with “No High School Diploma” (*n* = 1881). **b**–**d** Linear model fits (line fill ±95% CI) of the median svRT by Age^3^ (curvilinear model) with lines split by Handedness. **b** From 18–85 years old, left-handed participants showed slightly shorter svRTs (*β*_Left_ = −3.87, *p*_Left_ < 0.03), (**c**) an association found in 20–40 years old (*β*_40Left_ = −3.16, *p*_40Left_ < 0.01, Left-Handed *n* = 8,449), (**d**) but not 40–60 years old (*β*_60Left_ = −2.69, *p*_60Left_ = 0.07, Right-Handed *n* = 66,903).

Biological Sex was a significant predictor of svRT (*p*_Sex_ = 1.26E − 163). Being a man was associated with an average of 34 ms (9.63%) shorter svRT response than being a woman (Fig. 1d). Educational Attainment was also a significant factor associated with svRT. Compared to “No High School Diploma,” participants who had “Some College” (*p*_College_ = 8.74E − 05), or a “College Degree” (*p*_CDegree_ = 2.95E − 17, Fig. 2a) were shorter. Attending college and attaining a college degree was associated with a respective near 15 (4.14%) and 32 (8.92%) ms shorter svRT compared to not graduating from high school. Handedness was also associated with svRT. Left-handed participants had a near 4 ms (1.09%) shorter svRT (*p*_Left_ = 0.03, Fig. 2b). This association was present in individuals 20 to 40 years old (*p*_40Left_ < 0.01, Fig. 2c) but not in individuals 40 to 60 years old (*p*_60Left_ = 0.07, Fig. 2d).

### MindCrowd: health, medical, and lifestyle factors

**Fig. 3:**
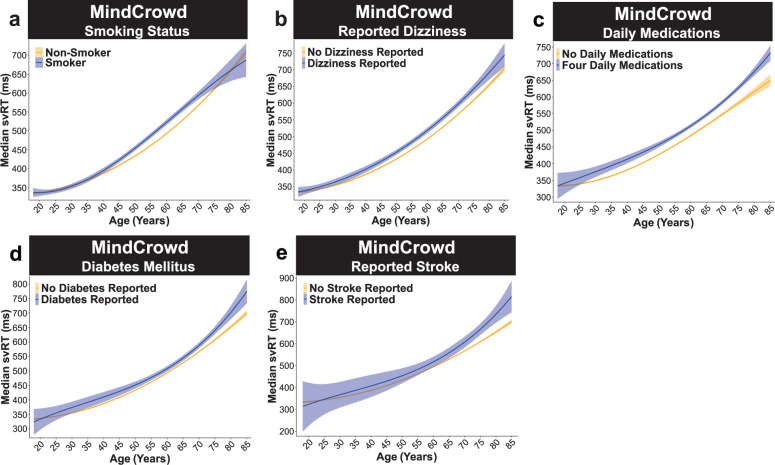
MindCrowd: health, medical, and lifestyle factors. MindCrowd analysis (ages 18–85) of simple visual reaction time (svRT). **a**–**b** Smoking Status and Reported Dizziness were associated with lengthened svRT. **a** Linear model fits (line fill ±95% CI) of the median svRT by Age^3^ (curvilinear model) with lines split by Smoking Status. Participants identifying as a smoker showed lengthened svRT (*β*_Smoking_ = 7.07, *p*_Smoking_ = 1.26E − 03, Smoker *n* = 5793, Non-Smoker *n* = 69,873). **b** Linear model fits (line fill ±95% CI) of the median svRT by Age^3^ (curvilinear model) with lines split by Reported Dizziness. Participants reporting Reported Dizziness showed lengthened svRT performance (*β*_Dizzy_ = 4.87, *p*_Dizzy_ = 0.04, Reported Dizziness Reported *n* = 4749, No Reported Dizziness Reported *n* = 70,917). **c**–**e** Daily Medications, Diabetes, and Reported Stroke were all associated with lengthened svRT. MindCrowd analysis (ages 18–85). **c** Linear model fits (line fill ±95% CI) of the median svRT by Age^3^ (curvilinear model) with lines split by Daily Medications taken. Compared to participants reporting “no daily medications” (*n* = 33,672), taking “Two” (*β*_Meds2_ = 5.84, *p*_Meds2_ = 2.00E − 03, *n* = 9651), “Three” (*β*_Meds3_ = 6.24, *p*_Meds3_ < 0.01, *n* = 6656), and “Four” (*β*_Meds4_ = 17.82, *p*_Meds4_ = 3.51E − 16, *n* = 10,769) lengthened svRT. **d** Linear model fits (line fill ±95% CI) of the median svRT by Age^3^ (curvilinear model) with lines split by Diabetes Mellitus. Participants reporting having diabetes were associated with lengthened svRT (*β*_Diabetes_ = 11.23, *p*_Diabetes_ < 3.36E − 05, Diabetes Reported *n* = 3887, No Diabetes Reported *n* = 71,779). **e** Linear model fits (line fill ±95% CI) of the median svRT by Age^3^ (curvilinear model) with lines split by Reported Stroke. A reported stroke was associated with longer svRTs (*β*_Stroke_ = 20.38, *p*_Stroke_ = 3.59E − 04, Reported Stroke *n* = 765, No Reported Stroke *n* = 74,901).

For health and medical factors associated with svRT, we found that Smoking Status (*p*_Smoking_ = 1.26E − 03, Fig. 3a) and Reported Dizziness (*p*_Dizzy_ = 0.04, Fig. 3b) were both significant predictors of svRT. Smoking Status was associated with 7 ms (1.99%) lengthened svRT, and Reported Dizziness was associated with nearly a 5 ms (1.37%) lengthened svRT. When compared to participants reporting “no daily medications,” taking “Two” (*p*_Meds2_ = 2.00E − 03), “Three” (*p*_*Meds3*_ < 0.01), and “Four” (*p*_Meds4_ = 3.51E − 16) Daily Medications were associated with an approximate 5 (1.64%), 6 (1.76%), and 18 (5.01%) ms longer svRTs, respectively (Fig. 3c). Further, Diabetes Mellitus (*p*_Diabetes_ < 3.36E − 05, Fig. 3d), and Stroke (*p*_Stroke_ = 3.59E − 04, Fig. 3e) were related to 11 (3.16%) and 20 (5.73%) ms longer svRTs, respectively, when compared to participants not reporting either condition. Of note, in this model, both a first-degree family history of Alzheimer’s disease (FHAD; *p*_FHAD_ = 0.78) and Hypertension (*p*_Hyper_ = 0.52) were not significant predictors of svRT performance (Supplementary Fig. 3A–B).

### MindCrowd: two-way interactions

For interactions, we found Age significantly interacted with PAL Performance. Age × PAL Performance (*p*_Age*PAL_ = 9.93E − 62). Analysis of simple slopes suggests that each word pair correct was associated with shorter svRT from younger to older ages. That is, at 20 (*p*_Age20*PAL_ < 0.00), 40 (*p*_Age40*PAL_ < 0.00), 60 (*p*_Age60*PAL_ < 0.00), and 80 (*p*_Age80*PAL_ < 0.00, Fig. 1c) years old. There was a significant Biological Sex × Age interaction (*p*_Age*Sex_ = 4.61E − 08), indicating that the associated slowing of svRT at younger and older ages in men, compared to women, was 0.36 ms (0.08%) less per one year difference in age (Fig. 1c). These data suggest that men’s age-associated svRT lengthening was slower when compared to women. Of interest, in both women and men, we found significant Age × Educational Attainment interactions. Compared to Age × “No High School Diploma”, participants reporting having “Some College” (*p*_Age*College_ = 4.20E − 04) or a College Degree” (*p*_Age*CDegre_ = 2.07E − 12) was associated with longer RTs from young to an older age. These results suggest that attending college or getting a college degree was associated with a 0.65 (0.15%) and 1.31 (0.30%) ms shortened svRT performance per one year difference in age, respectively (Fig. 2a). The MindCrowd model revealed a significant Age × Reported Stroke interaction (*p*_Age*Stroke_ = 2.72E − 06). Participants who Reported Stroke were associated with an approximate 2 ms (0.37%) longer svRT per a one-year difference in age (Fig. 3e). Lastly, we found a significant Age × Smoking Status interaction (*p*_Age*Smoke_ = 5.67E − 07). This interaction suggests that Smoking Status lengthens svRT by adding 0.57 ms (0.11%) per year difference in age (Fig. 3a). See Table 3 for a summary of MindCrowd’s results.

**Table 3:** Summary MindCrowd’s main results.

Factor	Value	*β*	SE	*t*	*p*
Intercept		355.88	11.99	29.68	1.89E − 192
Age^1^	One year difference (i.e., slope)	7.07	0.81	8.71	3.06E − 18
Age^2^	One year difference bend in the slope	−0.15	0.02	−8.44	3.23E − 17
Age^3^	One year difference in slope bend	1.20E − 04	0	12.27	1.46E − 34
PAL performance^1^	One word pair difference (i.e., slope)	−8.89	0.73	−12.19	3.77E − 34
PAL performance^2^	One word pair difference bend in slope	0.32	0.04	7.63	2.35E − 14
PAL performance^3^	Per word pair difference in slope bend	7.14E − 04	0	−5.78	7.28E − 09
Biological sex	Men	−34.26	1.25	−27.33	1.26E − 163
Educational attainment	Some college	−14.73	3.75	−3.92	8.74E − 05
Educational attainment	College degree	−31.78	3.76	−8.45	2.95E − 17
Handedness	Left	−3.87	1.76	−2.2	0.03
Daily medications taken	Two	5.84	1.89	3.09	2.00E − 03
Daily medications taken	Three	6.24	2.26	2.76	0.01
Daily medications taken	Four	17.82	2.18	8.16	3.51E − 16
Reported dizziness	True	4.87	2.35	2.07	0.04
Smoking status	True	7.07	2.19	3.23	1.26E − 03
Diabetes mellitus	True	11.23	2.71	4.15	3.36E − 05
Reported stroke	True	20.38	5.71	3.57	3.59E − 04
Age × PAL performance	Better PAL Performance shortens the age-associated shift in svRT	−7.56E − 04	4.41E − 05	−1.72E + 01	8.34E − 66
Age × smoking status	Smoking status lengthens the age-associated shift in svRT	0.58	0.12	4.67	2.95E − 06
Age × reported stroke	Reported stroke lengthens the age-associated shift in svRT	1.87	0.42	4.46	8.17E − 06
Age × biological sex	Men shift toward shorter svRT	−0.37	0.07	−5.59	2.23E − 08
Age × educational attainment	Having some college vs. No high school diploma shortens the age-associated shift in svRT	−0.76	0.18	−4.09	4.28E − 05
Age × educational attainment	A college degree vs. No high school diploma shortens the age-associated shift in svRT	−1.53	0.19	−8.14	4.08E − 16

List of MindCrowd’s main associations and interactions, *β* = unstandardized regression coefficient, *SE* = standard error, *t* = value of *t*-statistic, *p* = *p*-value.

Superscript numbers denotes the degree of the polynomial.

### MindCrowd: mobile device

Participants who were identified as using a mobile device to take MindCrowd (i.e., using a touchscreen, *n* = 7603, age *M* = 54.06 SD = 14.54 years) were associated with longer svRTs and were older (*β*_Age–Mobile_ = 14.13, *p*_Age–Mobile_ < 2e − 16)compared to those who did not use a mobile device (*n* = 76,775, age *M* = 45.54 SD = 18.43 years, see Supplementary Fig. 4).

### UKBb MindCrowd and UK Biobank

Of the total 75,666 MindCrowd participants, 39,759 between the ages of 40 and 70 were selected to mirror the UK Biobank. This subset is called UKBb MindCrowd from here on to differentiate it from MindCrowd. After filtering (see “Methods” section: Data Quality Control), the UK Biobank cohort had 158,249 participants, derived from a data request we received on 9–19–2019 (See Table 1 for Sociodemographic Characteristics and Supplementary Fig. 1B–C for age histograms). We model both the UKBb MindCrowd’s svRT (see Supplementary Fig. 5 for regression diagnostic plots) as well UK Biobank’s cvrRT (see Supplementary Fig. 6 for regression diagnostic plots) as a function of 11 shared survey questions (see Table 4 for MindCrowd and UK Biobank’s sample sizes [*n*s]). The omnibus UKBb MindCrowd (*F*_mcomni_[20, 38871] = 1039, *p*_mcomni_ < 2.2e − 16, Adjusted *R*^2^ = 0.08) and UK Biobank (*F*_ukbbbomni_[20, 157903] = 1038, *p*_ukbbomni_ < 2.2e−16, Adjusted *R*^2^ = 0.13) LMs were both significant. Table 5 summarizes the results from UKBb MindCrowd and the UK Biobank side by side.

**Table 4:** UKBb MindCrowd and UK Biobank’s sample size (ns) summary.

Characteristic	UKBb MindCrowd ns		UK Biobank ns
Biological sex	Women = 29,640	Women = 89,331	
	Men = 10,155	Men = 68,914	
Diabetes mellitus	Diabetes reported = 2807	Diabetes reported = 4969	
	No diabetes reported = 36,988	No diabetes reported = 153,276	
Handedness	Left-handed = 4520	Left-handed = 15,287	
	Right-handed = 35,034	Right-handed = 142,958	
Reported stroke	Reported stroke reported = 2807	Reported stroke reported = 1237	
	No reported stroke reported = 39,318	No reported stroke reported = 157,008	
Reported hypertension	Hypertension Rep. = 9676	Hypertension rep. = 32,593	
	No Hypertension Rep. = 30,119	No hypertension rep. = 125,652	
Smoking status	Smoker = 2783	Smoker = 91,312	
	Non-smoker = 37,012	Non-smoker = 66,923	
Reported dizziness	Reported dizziness reported = 2543	Reported dizziness reported = 42,210	
	No reported dizziness reported = 37,252	No reported dizziness reported = 116,035	
Educational attainment	No high school diploma = 605	No high school diploma = 10,978	
	High school diploma = 3176	High school diploma = 46,247	
	Some college = 11,139	Some college = 77,270	
	College degree = 24,875	College degree = 23,750	
FHAD	FHAD Reported = 13,748	FHAD reported = 19,741	
	No FHAD Reported = 26,047	No FHAD reported = 138,504	
Educational attainment × biological sex	Women	Women	
	No high school diploma = 426	No high school diploma = 6056	
	High school diploma = 2496	High school diploma = 27,742	
	Some college = 8740	Some college = 43,040	
	College degree = 17,978	College degree = 23,750	
	Men	Men	
	No high school diploma = 179	No high school diploma = 4922	
	High school diploma = 680	High school diploma = 18,505	
	Some college = 2399	Some college = 34,230	
	College degree = 12,493	College degree = 11,257	

List of the multiple regression coefficient (i.e., linear model factors) *n*s from UKBb MindCrowd and the UK Biobank.

**Table 5:** Summary of the key results from UKBb MindCrowd and the UK Biobank.

		UKBb MindCrowd				The UK Biobank				
Variable	Value	*β*	SE	*t*	*p*	*β*	SE	*t*	*p*	
Intercept		189.77	10.65	18.35	6.46E − 75	335.93	1.69	209.93	2.00E − 16	Agree
Age	Per year	5.75	0.12	46.57	2.00E − 16	3.40	0.03	132.87	2.00E − 16	Agree
Biological sex	Men	−40.00	2.24	−17.83	8.03E − 71	−18.28	0.38	−47.70	2.00E − 16	Agree
Diabetes mellitus	True	11.48	3.91	2.94	3.31E − 03	5.48	1.09	5.03	4.80E − 07	Agree
Handedness	Left	−2.58	3.05	−0.85	4.00E − 01	0.58	0.63	0.92	0.36	N.S. in both-but MindCrowd subtle effect
Reported stroke	True	18.47	9.02	2.05	4.00E − 02	10.61	2.13	4.99	6.15E − 07	Agree
Reported hypertension	True	6.99	2.37	2.95	3.16E − 03	1.09	0.48	2.27	2.00E − 02	Agree
Smoking status	True	10.18	3.87	2.63	0.01	0.40	0.38	1.05	0.29	MindCrowd Finding: Both Primary and UKBb
Reported dizziness	True	12.10	4.00	3.02	2.52E − 03	3.21	0.42	7.57	3.71E − 14	Agree
Educational attainment	High school diploma	−9.68	8.77	−1.10	2.70E − 01	−9.68	0.80	−12.09	1.23E − 33	Agree
Educational attainment	Some college	−26.08	8.27	−3.15	1.61E − 03	−10.26	0.77	−13.35	1.30E − 40	Agree
Educational attainment	College degree	−49.07	8.17	−6.01	1.91E − 09	−11.71	0.87	−13.51	1.46E − 41	Agree
First-degree family history of Alzheimer’s disease	True	−0.07	2.06	−0.04	0.97	2.43	0.57	4.26	2.04E − 05	UK Biobank Finding
Age × biological sex	Men’s vs. women’s per year change in Age slope	−0.62	0.26	−2.38	2.00E − 02	−0.55	0.05	−10.99	4.18E − 28	Agree
Biological sex by educational attainment	Men with high school diploma vs. women with no high school diploma	21.63	19.62	1.10	0.27	−11.24	1.61	−6.96	3.30E − 12	UK Biobank Finding
Biological sex by educational attainment	Men with some college vs. women with no high school diploma	16.25	18.28	0.89	0.37	−10.54	1.54	−6.83	8.71E − 12	UK Biobank Finding
Biological sex by educational attainment	Men with a college degree vs. women with no high school diploma	14.79	17.94	0.82	0.41	−12.8	1.74	−7.37	1.68E − 13	UK Biobank Finding

Displayed are factor names, factor definitions, *β* = unstandardized regression coefficients, as well as estimate *SE* = standard error, *t* = value of *t*-statistic, and *p* = *p*-value across both cohorts. The last column displays model outcomes in terms of the agreement between UKBb MindCrowd and the UK Biobank.

### UKBb MindCrowd and UK Biobank: age and sex

**Fig. 4:**
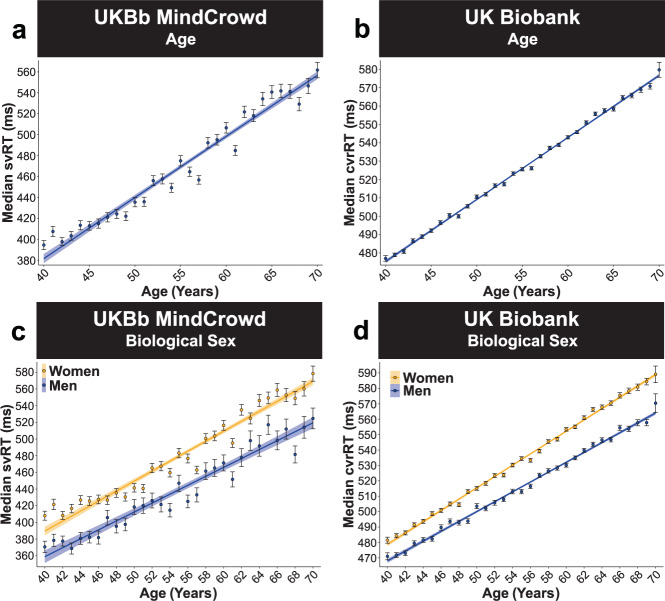
UK Biobank: age and biological sex. UKBb MindCrowd and UK Biobank analysis (ages 40–70) of visual reaction time (RT). **a**–**b** Age was linearly associated with RT. **a** UKBb MindCrowd linear model fits (line fill ±95% CI, error bars ± SEM) of median simple visual RT (svRT) from young to old Age. **b** UK Biobank linear model fits (line fill ±95% CI, error bars ± SEM) of median complex visual recognition RT (cvrRT) from young to old Age. UKBb MindCrowd svRT (*β*_Age_ = 5.75, *p*_Age_ = 2.00E − 16, *n* = 39,795) and UK Biobank cvrRT (*β*_Age_ = 3.40, *p*_Age_ = 2.00E − 16*, n* = 158,245) were associated with similar lengthening from younger to older ages. The average 50 ms difference between UKBb MindCrowd svRT (*M* = 478.66 ms) and UK Biobank (*M* = 528.74 ms) is due to the choice component (i.e., do cards match or not > press button) of the UK Biobank’s cvrRT task compared to UKBb MindCrowd’s stimulus-response (i.e., the pink sphere appears > press button) svRT. **c**–**d** Being a man, as compared to being a woman, was associated with shorter visual RT. **c** UKBb MindCrowd linear model fits (line fill ±95% CI, error bars ± SEM) of median svRT from young to old Age with lines split by Biological Sex. **d** UK Biobank linear model fits (line fill ±95% CI, error bars ± SEM) of median cvrRT from young to old Age with lines split by Biological Sex. Both UKBb MindCrowd svRT (*β*_Sex_ = −40.00, *p*_*Sex*_ = 8.03E − 71, 20.46%, Women [*M* = 489.75 ms, *n* = 29,640], Men [*M* = 446.28 ms, *n* = 10,155]) and UK Biobank cvrRT (*β*_*Sex*_ = −18.28, *p*_*Sex*_ = 2.00E − 16, 5.16%, Women [*M* = 534.98 ms*, n* = 89,331], Men [520.66 ms*, n* = 68,914) found that being a man was consistently associated with shorter RT when compared to being a woman from 40–70 years of age.

The UKBb MindCrowd cohort revealed Age as a significant predictor of svRT (*p*_Age_ = 2.00E − 16). The parallel analysis (see “Statistical Methods” section) of Age in the UK Biobank cohort was also significant (*p*_Age_ = 2.00E − 16) for complex visual recognition reaction time (cvrRT). For the association of Age and RT, UKBb MindCrowd and the UK Biobank showed longer RTs or worse RT performance from younger to older ages, with nearly 6 and 3 ms lengthened RT per year difference of age, respectively (Fig. 4a–b). For UKBb MindCrowd, Biological Sex was a significant predictor of RT (*β*_Sex_ = −40.00, *p*_Sex_ = 8.03E − 71), which was also the case in the UK Biobank (*β*_Sex_ = −18.28, *p*_Sex_ = 2.00E − 16). Being a man in both cohorts was associated with shorter RTs compared to being a woman (Fig. 4c–d). Here the effect of Biological Sex on RT between UKBb MindCrowd was 40 ms (20.46%), and the UK Biobank was 18 ms (5.16%).

### UKBb MindCrowd and UK Biobank: education and handedness

**Fig. 5:**
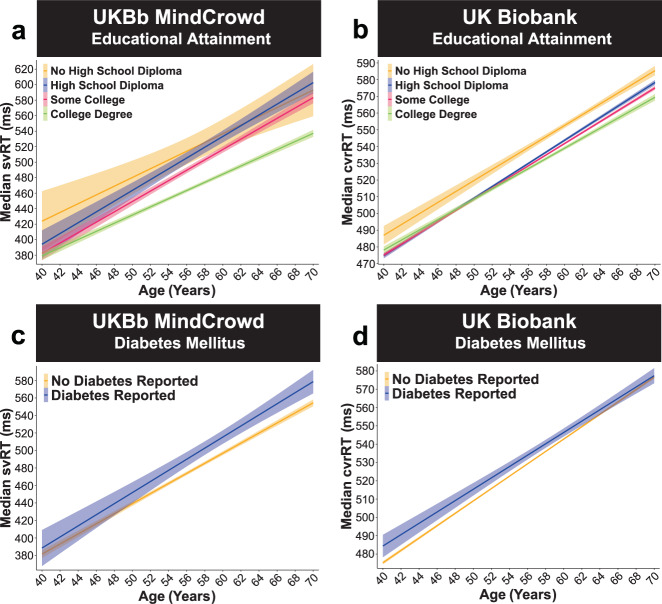
UK Biobank: educational attainment and diabetes mellitus. UKBb MindCrowd and UK Biobank analysis (ages 40–70) of visual reaction time (RT). **a**–**b** More education was related to shorter visual RT. **a** UKBb MindCrowd linear model fits (line fill ±95% CI) of median simple visual RT (svRT) from young to old Age with lines split by Educational Attainment. Participants who had a “High School Diploma” (*β*_HSDiploma_ = −9.68, *p*_HSDiploma_ = 2.70E − 01, 1.87%*, n* = 3176), “Some College” (*β*_College_ = −26.08, *p*_College_ = 1.61E − 03, 5.06%, *n* = 11,139), or a “College Degree” (*β*_CDegree_ = −49.07, *p*_CDegree_ = 1.91E − 09, 9.51%, *n* = 24,875) were shortened than those with “No High School Diploma” (*n* = 605). **b** UK Biobank linear model fits (line fill ±95% CI) of median complex visual recognition RT (cvrRT) from young to old Age with lines split by Educational Attainment. Like the UKBb MindCrowd cohort, participants who had a “High School Diploma” (*β*_HSDiploma_ = −9.68, *p*_HSDiploma_ = 1.23E − 33, 1.74%*, n* = 46,247), “Some College” (*β*_College_ = −10.26, *p*_*College*_ = 1.30E − 40, 1.85%, *n* = 77,270), or a “College Degree” (*β*_CDegree_ = −11.71, *p*_CDegree_ = 1.46E − 41, 2.11%, *n* = 23,750) were all associated with shorter cvrRTs when compared to “No High School Diploma” (*n* = 10,978). **c**–**d** Diabetes Mellitus was associated with lengthened visual RT. **c** UKBb MindCrowd linear model fits (line fill ±95% CI) of median svRT from young to old Age with lines split by diabetes mellitus. **d** UK Biobank linear model fits (line fill ±95% CI) of median cvrRT from young to old Age with lines split by diabetes mellitus. For the UKBb MindCrowd, individuals who reported (*β*_Diabetes_ = 11.48, *p*_Diabetes_ = 3.31E − 03, Diabetes Reported *n* = 2807, No Diabetes Reported *n* = 36,988) and UK Biobank cvrRT (*β*_Diabetes_ = 5.48, *p*_Diabetes_ = 4.80E − 07, Diabetes Reported *n* = 4969, No Diabetes Reported *n* = 153,276), were associated with lengthened svRT.

Akin to the association found in the MindCrowd analysis, the UKBb MindCrowd and UK Biobank comparison found that Educational Attainment was a significant RT predictor. Indeed, for UKBb MindCrowd a “High School Diploma” (*β*_HSDiploma_ = −9.68, *p*_HSDiploma_ = 2.70E − 01, 1.87%, “Some College” (*β*_College_ = −26.08, *p*_College_ = 1.61E − 03, 5.06%) and a “College Degree” (*β*_CDegree_ = −49.07, *p*_CDegree_ = 1.91E − 09, 9.51%), and in the UK Biobank a “High School Diploma” (*β*_HSDiploma_ = −9.68, *p*_HSDiploma_ = 1.23E − 33, 1.74%), “Some College” (*β*_College_ = −10.26, *p*_College_ = 1.30E − 40, 1.85%), or a “College Degree” (*β*_CDegree_ = −11.71, *p*_CDegree_ = 1.46E − 41, 2.11%) were all significantly different from “No High School Diploma” (Fig. 5a–b). Here, both UKBb MindCrowd and UK Biobank large cohorts reported shorter RT was associated with more education. Lastly, unlike the MindCrowd analyses, both the UKBb MindCrowd *(p*_Handedness_ = .40) and the UK Biobank (*p*_Handedness_ = 0.36) cohorts between the ages of 40–70 did not find Handedness to be a significant predictor of RT performance (Supplementary Fig. 7).

### UKBb MindCrowd and UK Biobank: health, medical, and lifestyle factors

**Fig. 6:**
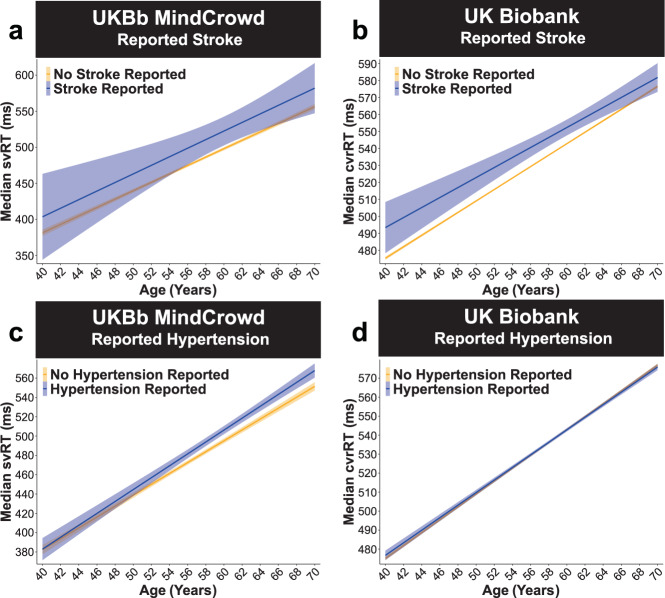
UK Biobank: reported stroke and hypertension. UKBb MindCrowd and UK Biobank analysis (ages 40–70) of visual reaction time (RT). **a**–**b** Reported Stroke was associated with lengthened visual reaction time (RT). **a** UKBb MindCrowd linear model fits (line fill ±95% CI) of median simple visual RT (svRT) from young to old Age with lines split by Reported Stroke. **b** UK Biobank linear model fits (line fill ±95% CI) of median complex visual recognition RT (cvrRT) from young to old Age with lines split by Reported Stroke. In both the UKBb MindCrowd svRT (*β*_Stroke_ = 18.47, *p*_Stroke_ = 4.00E − 02, Reported Stroke *n* = 2807, No Reported Stroke *n* = 39,318) and UK Biobank cvrRT (*β*_Stroke_ = 10.61, *p*_Stroke_ = 6.15E − 07, Reported Stroke *n* = 1237, No Reported Stroke *n* = 157,008) analysis, experiencing a Reported Stroke was associated with lengthened visual RT. **c**–**d** Reported Hypertension was associated with lengthened visual RT. **c** UKBb MindCrowd linear model fits (line fill ±95% CI) of median svRT from young to old Age with lines split by Reported Hypertension. **d** UK Biobank linear model fits (line fill ±95% CI) of median cvrRT from young to old Age with lines split by Reported Hypertension. Unlike the MindCrowd analysis, hypertension was related to longer svRTs in UKBb MindCrowd (*β*_Hypertension_ = 7.99, *p*_Hypertension_ = 3.16E − 03, Hypertension Reported *n* = 9676, No Hypertension Reported *n* = 30,119) and cvrRT in the UK Biobank (*β*_Hypertension_ = 1.14, *p*_Hypertension_ = 0.02, Hypertension Reported *n* = 32,593, No Hypertension Reported *n* = 125,652).

**Fig. 7:**
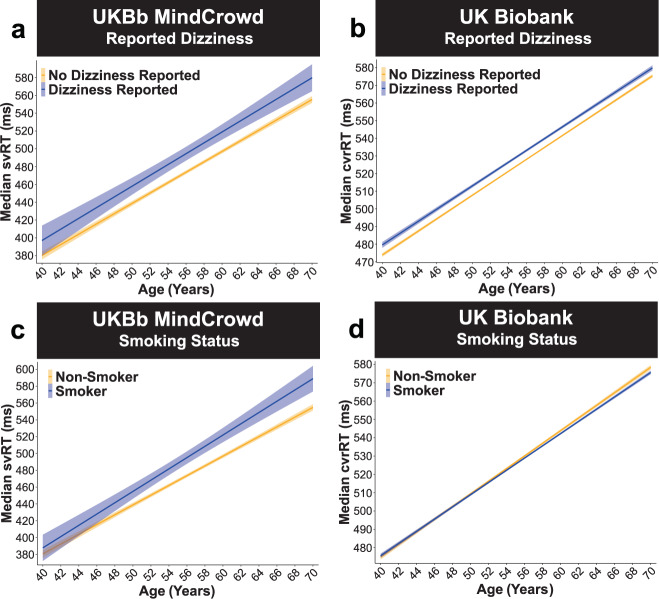
UK Biobank: reported dizziness and smoking status. UKBb MindCrowd and UK Biobank analysis (ages 40–70) of visual reaction time (RT). **a**–**b** Reported Dizziness was associated with lengthened visual RT. **a** UKBb MindCrowd linear model fits (line fill ±95% CI) of median simple visual RT (svRT) from young to old Age with lines split by Reported Dizziness. **b** UK Biobank linear model fits (line fill ±95% CI) of median complex visual recognition RT (cvrRT) from young to old Age with lines split by Reported Dizziness. UKBb MindCrowd svRT (*β*_Dizzy_ = 12.13, *p*_Dizzy_ = 2.52E − 03, Reported Dizziness Reported *n* = 2543, No Reported Dizziness Reported *n* = 37,252) and UK Biobank cvrRT (*β*_Dizzy_ = 3.21, *p*_Dizzy_ = 3.71E − 14, Reported Dizziness Reported *n* = 42,210, No Reported Dizziness Reported *n* = 116,035) were lengthened if participants Reported Dizziness. **c**–**d** Smoking Status was associated with lengthened svRT in UKBb MindCrowd, but not cvrRT in the UK Biobank. **c** UKBb MindCrowd linear model fits (line fill ±95% CI) of median svRT from young to old Age with lines split by Smoking Status. Compared to non-smokers, smokers were associated with longer svRTs (*β*_Smoke_ = 10.18, *p*_Smoke_ = 0.01, Smoker *n* = 2783, Non-Smoker *n* = 37,012). **d** UK Biobank linear model fits (line fill ±95% CI) of median cvrRT from young to old Age with lines split by Smoking Status. An association between Smoking Status and cvrRT was not found in the UK Biobank (*β*_Smoke_ = 0.47, *p*_Smoke_ = 0.22, Smoker *n* = 91,312, Non-Smoker *n* = 66,923).

**Fig. 8:**
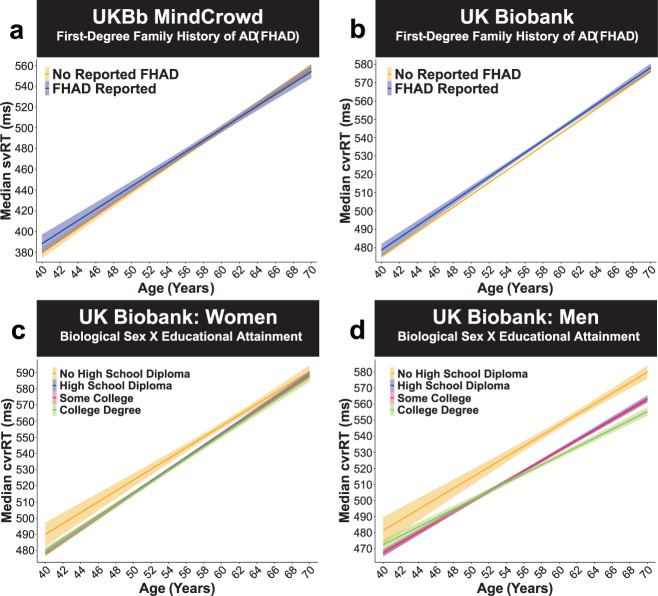
UK Biobank: FHAD biological and sex × educational attainment. UKBb MindCrowd and UK Biobank analysis (ages 40–70) of visual reaction time (RT). **a**–**b** A first-degree family history of Alzheimer’s disease (FHAD) was related to longer complex visual recognition reaction time (cvrRT)s in the UK Biobank, but not simple visual reaction time (svRT) in UKBb MindCrowd. **a** UKBb MindCrowd linear model fits (line fill ±95% CI) of median svRT from young to old Age with lines split by reported FHAD. An association between FHAD and svRT was not found in the UKBb MindCrowd cohort (*β*_FHAD_ = −0.07, *p*_FHAD_ = 0.97, FHAD Reported *n* = 13,748, No FHAD Reported *n* = 26,047). **b** UK Biobank linear model fits (line fill ±95% CI) of median cvrRT from young to old Age with lines split by reported FHAD. Compared to those reporting No FHAD, FHAD was related to worse cvrRT performance in the UK Biobank (*β*_FHAD_ = 2.36, *p*_FHAD_ = 3.35E − 05, FHAD Reported *n* = 19,741, No FHAD Reported *n* = 138,504). **c**–**d** In the UK Biobank, Biological Sex modified the association of Educational Attainment on cvrRT (Biological Sex × Educational Attainment interaction). Linear model fits (line fill ±95% CI) of the median cvrRT by Age with lines split by Educational Attainment in **c** women and (**d**) men. Compared to **c** women having “No High School Diploma” (*n* = 6056), (**d**) men with a “High School Diploma” (*β*_Sex*HSDiploma_ = −11.24, *p*_Sex*HSDiploma_ = 3.30E − 12, *n* = 18,505), “Some College” (*β*_sex*College_ = −10.54, *p*_sex*College_ = 8.71E − 12, *n* = 34,230) or a “College Degree” (*β*_sex*CDegree_ = −12.8, *p*_sex*CDegre_ = 1.68E − 13, *n* = 11,257) were associated with shortened cvrRT. See Supplementary Fig. 8, displaying simple effects parsed using estimated marginal means (EMM).

In terms of health factors associated with RT, in the UKBb MindCrowd cohort, Diabetes (*β*_Diabetes_ = 11.48, *p*_Diabetes_ = 3.31E − 03, 5.87%), Stroke (*β*_Stroke_ = 18.47, *p*_Stroke_ = 4.00E − 02, 9.45%), (*β*_Hypertension_ = 7.99, *p*_Hypertension_ = 3.16E − 03, 3.58%), and Dizziness (*β*_Dizzy_ = 12.13, *p*_Dizzy_ = 2.52E − 03, 6.19%) were all significantly associated with longer svRTs. These associations were recapitulated by the UK Biobank. To that end, Diabetes Mellitus (*β*_Diabetes_ = 5.48, *p*_Diabetes_ = 4.80E − 07, 1.55%, Fig. 5c–d), Reported Stroke (*β*_Stroke_ = 10.61, *p*_Stroke_ = 6.15E − 07, 2.99%, Fig. 6a–b), Reported Hypertension (*β*_Hypertension_ = 1.14, *p*_Hypertension_ = 0.02, 0.31%, Fig. 6c–d), and Reported Dizziness (*β*_Dizzy_ = 3.21, *p*_Dizzy_ = 3.71E − 14, 0.91%, Fig. 7a–b) were all significantly related to longer cvrRTs; however, the association between Reported Hypertension and cvrRT was small (Fig. 6d). Further, in agreement with the MindCrowd analysis, Smoking Status was significantly related to svRT (*β*_Smoke_ = 10.18, *p*_Smoke_ = 0.01, 5.21%) in the UKBb MindCrowd cohort; however, FHAD (*β*_FHAD_ = −0.07, *p*_FHAD_ = 0.97) was not. This pattern of associations was reversed in the UK Biobank; that is, Smoking Status was not a significant predictor of cvrRT (*β*_Smoke_ = 0.47, *p*_Smoke_ = 0.22, Fig. 7c–d), but FHAD was (*β*_FHAD_ = 2.36, *p*_FHAD_ = 3.35E − 05, 0.69%, Fig. 8a–b).

### UKBb MindCrowd and UK Biobank: two-way interactions

The “glmulti”^35^ R package defined two interactions in the UKBb MindCrowd and UK Biobank analysis. In UKBb MindCrowd we found a significant Age × Biological Sex interaction (*p*_Age*Sex_ = 2.00E − 02, 0.33%). We found a comparable significant Age × Biological Sex interaction for cvrRT (*p*_Age*Sex_ = 4.18E − 28, 0.16%) in the UK Biobank. Across both MindCrowd and the UK Biobank, these interactions indicated that RT was lengthened in men from younger to older ages compared to women, was over 0.5 ms shorter per one year difference in age (Fig. 4c–d). In addition, the UK Biobank analysis revealed a significant Biological Sex × Educational Attainment interaction not found in UKBb MindCrowd. Here, men with a “High School Diploma” (*p*_Sex*HSDiploma_ = 3.30E − 12, 3.35%), “Some College” (*p*_sex*College_ = 8.71E − 12, 3.14%) or a “College Degree” (*p*_sex*CDegre_ = 1.68E − 13, 3.18%) were significantly associated with shorter cvrRT performance when compared to women having “No High School Diploma” (Fig. 8c). Follow-up analyses of the simple effects via estimated marginal means (EMM, see “Statistical Methods” section) revealed that men who did not graduate high school (EMM = 558.91 ms), compared to men with more education (EMMs = 542.99, 542.98, and 540.41 ms), had markedly shortened cvrRTs, more in line with the women’s cvrRT performance (EMMs = 566.80, 562.11, 561.40, and 561.09 ms). The associated difference in cvrRT for men with “No High School Diploma” compared to men with a “High School Diploma” (*β*_Men_ = 15.92, *p*_Men_ = 2.00E − 16, 1.75%) was more substantial than between women with “No High School Diploma” compared to women with a “High School Diploma” (*β*_Women_ = 4.68, *p*_Women_ = 3.12E − 04, 1.69%, see Supplementary Fig. 8 for a graph of the EMM).

## Discussion

**Fig. 9:**
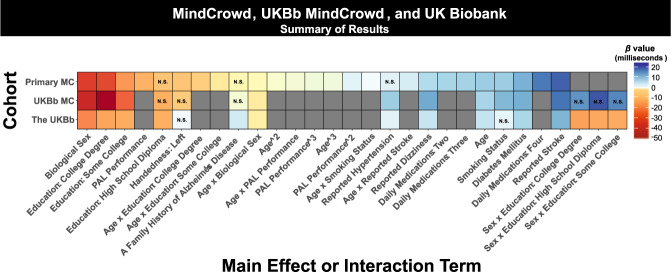
An illustrative summary of the overall results. Data are shown across the MindCrowd (MC), UKBb MindCrowd, and the UK Biobank (UKBb). The color (i.e., red = low negative and blue = high positive) indicates the size of the *β* (beta coefficient) estimate.” N.S.,” indicates if the estimated *β* value was not statistically significant (*α* = 0.05).

Our study’s results illuminate a portion of the intricate relationship between age and RT performance by identifying demographic, health, medical, and lifestyle factors associated with either attenuation or exacerbation of RT lengthening from younger to older ages (see Fig. 9 for an illustrative summary of the results). A large body of work on RT, leading back to Sir Francis Galton in 1890^36^, has consistently demonstrated an age-associated shift in RT^37^. It is not surprising that MindCrowd, UKBb MindCrowd, and UK Biobank models revealed slowing of simple visual (svRT, MindCrowd) and complex visual recognition RT (cvrRT, UK Biobank) from younger to older ages. Likewise, the MindCrowd model found that the relationship between svRT and age was modestly curvilinear (Fig. 1a). While this curvilinear relation between RT and age has been noted previously^38^, both cohorts’ large sample size combined with our application of an algorithm-based model definition^35^ revealed a notable addition to this picture. Specifically, in the MindCrowd analysis, we observed an interaction between age and education (Fig. 2a) and smoking (Fig. 3a). Here, less education and smoking were related to the additional slowing of simple visual RT (svRT) on top of the svRT slowing associated with transitioning from younger to older ages. Lastly, the age by reported stroke interaction modeled in MindCrowd was associated with longer RTs (Fig. 3e). This study’s large sample size and broad age and surveyed data range places it as one of the most substantial cross-sectional RT evaluations across the aging spectrum. Our findings suggest that smoking and stroke (i.e., cardiovascular health) and amount of education (i.e., cognitive demand or reserve) are factors, modifiable across aging, that influence age-associated RT slowing.

In the UK Biobank cohort^27^, we found an association between having an FHAD and lengthened (2.43 ms) cvrRT (Fig. 8b). This effect of FHAD on RT or more so underlying process speed is in line with our episodic verbal memory task (i.e., paired-associate learning [PAL]) finding^34^, where we found FHAD was linked to lower PAL performance. Furthermore, a prior functional magnetic resonance imaging (fMRI) study examining medial-temporal lobe activation using a cvrRT task found a ~100 ms RT lengthening in 68 (mean age of 54) FHAD participants^39^. These data suggest that genetic and environmental factors relating to AD risk are present in individuals with an FHAD. Indeed, the first-degree relatives of identified FHAD participants consisted of familial early-onset AD or late-onset AD, which also has a high heritability of 79%, suggesting that they are a higher risk category for developing AD. Thus, such shared AD and FHAD factors may relate to sensorimotor function and processing speed (i.e., RT) analogous to alterations in cognition and memory (i.e., PAL).

We found a correlation between svRT and PAL performance (Fig. 1b). This finding was in line with many prior studies, the dependence of episodic memory on processing speed, a dependence that grows with age and incident of age-related disease (e.g., AD)^5–8,40^. The association of svRT with PAL may highlight distributed systems and networks that underlie RT performance. For example, svRT performance could depend on the functioning of many cognitive areas that PAL requires and vice versa. Another possibility is that properly functioning memory and cognitive networks correspond to better RT performance. Evidence for this is suggested by the fact that higher intelligence is related to shorter RT^41–43^. However, diverse factors (e.g., exercise, time of day, meal proximity, and individual assessing RT) affect RT performance^40,43,44^. Thus, making an accurate estimation of RT’s effect on intelligence and vice versa changeling. Together with our prior results, these findings suggest that RT performance could be used as a metric to assess potential AD risk. However, further research, including longitudinal studies, replication, and corroboration of RT’s link to age-related cognitive decline and disease, are necessary to support this notion.

The difference in the effects of FHAD between UK Biobank and MindCrowd (for both MindCrowd and UKBb MindCrowd analyses, Supplementary Fig. 3A and Fig. 8a) could be due to the vast difference in the fraction of FHAD participants in the UK Biobank (FHAD = 13% of total) compared to UKBb MindCrowd (FHAD = 35% of total). While noting that we target those with FHAD for recruitment into MindCrowd, this substantial disparity could also be due to the accuracy of the UK Biobank’s FHAD. The UK Biobank was calculated from three separate questions (i.e., mother, father, and siblings AD diagnosis). Adding to this, the United Kingdom uses a massive, detailed, nationwide electronic health record system facilitating respondent health and medical survey accuracy. Compare this to our single question in MindCrowd, asking participants of all ages to remember if a relative was diagnosed with AD. Another possibility is the RT paradigm used; that is, MindCrowd’s test of svRT compared to the UK Biobank’s use of cvrRT. The fact that complex reaction time, requiring recognition and the choice to “respond or not respond,” rather than just stimulus-response, may underly this difference. UKBb MindCrowd svRT performance showed a consistently shorter association from young to old age compared to the UK Biobank cvrRT performance; an observation noted in a prior study also measured both simple and complex RT^45^. These findings are consistent with the idea that complex RT requires more processing time^12,46^, and prior work found the age-associated slowing of RT was higher for choice RT than simple RT^47^. Lastly, the differences in FHAD associations across MindCrowd and the UK Biobank are perhaps due to the UK Biobank had over 2× the number of participants compared to MindCrowd (i.e., 158 K vs. 76 K). The larger sample is expected to produce better model definitions and increased statistical power. Increased statistical power may also have enhanced accuracy, validity, and reproducibility.

Numerous RT studies have found sex differences in RT performance, which does not appear to be reduced by practice^16,20,45^. Consistent with others^45,47^, men exhibited shorter RTs in each model across cohorts (Figs. 1d and 4c–d). In addition, the analysis of the UKBb MindCrowd and UK Biobank implicated biological sex affecting RT slowing from younger to older ages. The age interaction with biological sex suggests that being a woman from younger to older ages is associated with longer RT compared to being a man. These results essentially replicate a previous sizable study (i.e., 7000 participants) evaluating RT^45^. Similar to our own, this study found that (1) men consistently outperformed women on all RT measures from younger to older ages, (2) differences in RT performance from younger to older ages were nonlinear, (3) including a third-degree polynomial for age provided the best model fit, and (4) compared to men, women displayed longer RTs consistently from younger to older ages^45^. Collected with our prior study of PAL performance^34^, these associations replicate prior work and suggest that biological sex affects RT and age-associated shift in RT.

Educational attainment was associated with svRT in both MindCrowd cohorts and cvrRT in the UK Biobank. Overall, having more education (i.e., reporting higher milestones) was related to shorter svRT and cvrRT (Figs. 2a and 5a–b). However, it is unclear if individuals with higher processing speed naturally seek more education and what other factors confound this relationship. Further work utilizing both cohorts is necessary to shed light on the effects and modifiers of FHAD and cross-cohort discrepancies. The model of the UK Biobank revealed an interaction between biological sex and education on RT performance. The breakdown revealed that men had similar RT performance if they attained a high school diploma and above. However, men who did not graduate high school showed markedly longer RTs, which brought them in line with women’s RT performance. However, the associated lengthening of RT for less-educated men was vastly more than that found in less-educated women (reported “No High School Diploma” made up 3.83% of women and 3.11% of men; see Supplementary Fig. 8).

In MindCrowd, handedness, specifically being left-handed, was associated with shorter svRTs. Prior studies have reported similar associations, where left-handedness was correlated with shorter svRT^19,46,48^. Hemispheric asymmetries in spatial processing are thought to underly shortened svRT for the left hand^47,49^. Handedness was not associated with svRT in UKBb MindCrowd or cvrRT in the UK Biobank. One explanation for the divergent findings is that MindCrowd includes younger participants (i.e., 18–40-year-olds). Indeed, in MindCrowd, the association appears to diminish from younger to older ages. Specifically, in Fig. 2b, the separation of the regression lines between left-handed and right-handed participants shrinks and eventually crosses around the 4th decade of life. Figure 2c shows that the left-handed and right-handed regression lines separate in 20 to 40-years-olds, while Fig. 2d shows that these regression lines are not separate in 40 to 60-year-olds. While purely speculative, differences in social conventions may have played a role. For example, some older participants were forced to be right-handed, whereas younger participants were not. In doing so, upping the amount of unexplained variance in older, but not younger, participants across MindCrowd and the UK Biobank.

The MindCrowd analysis incorporated all 13 available health, medical, and lifestyle-related factors, of which six were present and incorporated into the shared UKBb MindCrowd/UK Biobank model (Tables 2 and 4). Before the launch of MindCrowd, these factors were carefully selected based upon their known relation to (1) age-associated alterations, (2) RT performance, and (3) PAL Performance. Of the 13 health, medical, and lifestyle factors evaluated in the MindCrowd analysis, we found associations between svRT and the number of daily medications, reported dizziness, smoking status, reported stroke, and diabetes mellitus. Each health and medical factor were associated with longer svRTs (Fig. 3). We should note that the number of daily medications is a serving as a proxy for overall health. That is, the worse one’s health, the worse one’s performance, the increased number of medications treating the underlying health conditions. The UKBb MindCrowd (svRT) and the UK Biobank (cvrRT) analyses found *similar* associations between reported dizziness, reported stroke, diabetes mellitus, indicating hypertension. Although each association differed in magnitude between the two older cohorts, each was related to lengthened RT. The UKBb MindCrowd to the UK Biobank found a different association for FHAD (UKBb MindCrowd = no association; UK Biobank = 2.43 ms longer), smoking status (UKBb MindCrowd = 10 ms longer; UK Biobank = no association). Interestingly, despite some differences, only a few coefficient signs differed between the UKBb MindCrowd and UK Biobank; indeed, most estimations were well within an order of magnitude between the two cohorts (e.g., age, educational attainment, and age by biological sex interaction, Table 5).

Many factors are likely to account for the different associations between smoking and FHAD between UKBb MindCrowd and the UK Biobank (Figs. 7c–d and 8a–b). Some of these include differences in demographics, genetic heterogeneity, and age^26^. However, candidates include the fractions of participants reporting each factor (e.g., for diabetes mellitus: MindCrowd = 1%, UKBb MindCrowd = 7%, and UK Biobank = 3%). Another factor is that the UK Biobank’s participant number is twice the size of MindCrowd and four times the size of UKBb MindCrowd. Despite our study’s size, the observational and cross-sectional method means that we cannot rule out effects due to confounding variables.

Consequently, while numbers may be close, we do not assume that the UKBb MindCrowd is similar and can be compared to the UK Biobank. Furthermore, we observed that UKBb MindCrowd consistently reported larger estimates and standard errors than the UK Biobank. For example, the MindCrowd cohort’s estimation of the sex difference association was consistently more extended (~40 ms), even in the UKBb MindCrowd cohort when looking at the UK Biobank (~19 ms). This difference demonstrates why the study of neuropsychological traits and disease requires large sizes to provide accurate estimations driving better predictive validity.

We strongly advocate for large-scale efforts like ours, the UK Biobank^50^, and others^22^. Indeed, studies of this kind have characteristics that provide the unique impact necessary to move the fields of aging and age-related diseases forward. These include: (1) statistical control, as our MindCrowd analysis incorporated all 24 available factors, 11 of which were used in the UKBb MindCrowd and UK Biobank model. (2) The inclusion of each predictor controlled for its association on RT, which potentially removed variability (noise), thus enhancing statistical power. (3) The two models used for each of the analyses were selected with little human input by automated application of specific statistical criteria (see Inclusion of polynomials and automatic model selection in “Statistical Methods” section). This likely decreases bias, the probability of overfitting, and multicollinearity. (4) For this study, MindCrowd had over 76 K and the UK Biobank over 158 K participants. Large sample sizes in each cohort were expected to help reduce variance, enhance estimation, select better models, and in turn, enhance statistical power. Expanded statistical power may then enhance accuracy, validity, and reproducibility.

Lastly, a recent genome-wide association study examining associations between RT and single nucleotide polymorphisms (SNP) in the UK Biobank and CHARGE and COGENT consortia noted weak correlations between the reported cognitive-associated SNPs among US and UK cohorts^51^. Here, MindCrowd presents a future opportunity to resolve these weak associations and get a better picture of potential cohort effects. Taken together, these characteristics increase the likelihood of making accurate inferences regarding associations while boosting predictive validity. These are both necessary and vital attributes when searching for genetic associations and the structure underlying healthy brain aging.

There are potential concerns that arise from web-based studies^52^. Indeed, limitations of this study include the cross-sectional design and the partial discrepancy in MindCrowd’s svRT test compared to the UK Biobank’s cvrRT test and info collected between the UK Biobank and MindCrowd (e.g., the omission of “prefer not to answer” choices for race and education questions). Acknowledging these drawbacks, we believe that the advantage of meaningfully higher participant numbers and enriched cohort diversity facilitated via online research remediates some disadvantages. For example, the range of error reported in recent internet-based studies of self-reported quantitative traits like height and weight was between 0.3 and 20%^53–56^. Previously, we ran simulations on the association between FHAD and PAL by randomly shuffling the FHAD responses (e.g., Yes to No, and No to Yes), introducing increasing sequential amounts of “error.” We found that even with a subtle effect such as FHAD on PAL performance, due to our cohort size, 24% error would still have only made us commit a Type1 error 50% of the time^34^. In line with this notion, another publication demonstrated that online RT studies produce reproducible results^57^.

Further, we developed an extensive and automated data filtering pipeline (see Data Quality Control and Supplementary Figs. 9–10) to address these concerns and enhance validity and accuracy. These data (i.e., raw or filtered) were excluded before analysis (i.e., listwise deletion). Exclusion resulted in dropping 0.3% and 6.1% of MindCrowd and UK Biobank participants, respectively. One of the 25 critical factors had over 5% missing data (see Supplementary Fig. 11 and Supplementary Tables 1 and 2). Reported Dizziness in the UK Biobank had 64.36% missing data. Hence, interpretation of this factor’s association with cvrRT should only be considered for “hypothesis-generation^58^.” Evaluation of selection bias between retained and excluded participants revealed an overall lower probability of exclusion in MindCrowd and higher likelihood in the UK Biobank (see Supplementary Tables 3 and 4). Notable groups with a higher probability of exclusion included those in the highest age ranges and those reporting hypertension and dizziness. These higher probability groups were found in both study’s cohorts.

Lastly, it is essential to note that our internet-based svRT task was not designed to directly mirror conventional face-to-face RT testing paradigms. Indeed, we find higher RTs and steeper slopes from younger to older ages than studies assessing svRT via the gold standard, laboratory-based assessments (e.g., refs. ^47,59^). However, these paradigm differences are not likely to alter our svRT test’s validity or reliability. One reason being our test is only interpreted within MindCrowd to identify associated factors and reveal individual differences. Despite test paradigm differences, we believe that large cross-sectional studies like MindCrowd, utilizing internet-based testing and remote biosample collection, are vital to moving the field of aging and age-related disease forward (see Opportunities: Unique impact above and^50^).

Understanding the modifiable and non-modifiable variables associated with RT and related cognitive function will begin to deconstruct the underlying architecture of elements accounting for the vast heterogeneity seen in individual trajectories of age-associated cognitive decline. Only then will it be possible to develop a healthy brain aging model that is both valid and reliable^60^. Such a model holds immense potential to attenuate age-related and disease-related cognitive deficits, thus enhancing cognitive healthspan. Any extension of cognitive healthspan, better aligning it to the human lifespan, would be invaluable and increasingly vital when aggregated across the aging population. Mitigating age-related or disease-related cognitive decline, allowing maintenance of independence by even only a few years, would have many benefits. For example, the U.S. could save billions of dollars in health care costs and lost caregivers’ productivity while improving the quality of life for the aging population^50^. In this study, we revealed several potential factors related to aging and processing speed. Of those, smoking and education, as potentially modifiable factors throughout life, were associated with longer and shorter RTs, respectively, from younger to older ages. With MindCrowd recruitment ever-increasing, our goal is to continue supplying and refining the knowledge necessary to optimize cognitive performance throughout life.

## Methods

### Study participants MindCrowd: overview

In January 2013, we launched our internet-based study at www.mindcrowd.org. Website visitors 18 years or older were asked to consent to our study before any data collection via an electronic consent form. As of 3–17–2020, we have had 356,674 non-duplicate or distinct visitors to the website. Of these distinct visitors, over 194,542 (54%) consented to take part. The final data set had 75,666 (39% of consented individuals) participants who completed a simple visual reaction time (svRT) and paired-associate learning (PAL) tasks and answered 22 demographic, lifestyle, and health questions. The authors confirm they obtained informed consent from each participant and complied with all relevant ethical regulations. Approval for this study was obtained from the Western Institutional Review Board (WIRB study number 1129241).

### Study participants MindCrowd: simple visual reaction time (svRT)

After consenting to the study and answering five demographic questions (i.e., age, biological sex, years of education, primary language, and country where they reside), participants were asked to complete a web-based svRT task. We chose svRT because it is a simple central and peripheral nervous system-dependent task influenced by intelligence and brain injury^61^. Participants were presented with a pink sphere that appeared at random intervals (between 1 s and 10 s) on the screen, and they were instructed to respond as quickly as possible after the sphere appeared by pressing the enter/return key on their keyboard. Once the participant responded, the sphere disappeared until the subsequent trial. Each participant received a total of five trials. The sphere stayed on the screen until the participant responded. The dependent variable, response time in milliseconds (ms), was recorded from the sphere’s appearance on the screen to the participant’s key press or screen touch.

### Study participants MindCrowd: paired-associate learning (PAL)

Next, participants were presented with the PAL task. For this cognitive task, during the learning phase, participants were shown 12-word pairs, one-word pair at a time (2 s/word pair). During the recall phase, participants were given the first word of each pair and were asked to use their keyboard to type in (i.e., recall) the missing word. This learning-recall procedure was repeated for two more trials. Before beginning the task, each participant received one practice trial consisting of three-word pairs not contained in the 12 used during the test. Word pairs were presented in different random orders during each learning and each recall phase. The same word pairs and order of presentation were used for all participants. The dependent variable/criterion was the total number of correct word pairs entered across the three trials (i.e., 12 × 3 = 36, a perfect score).

### Study participants MindCrowd: demographic, medical, health, and lifestyle questions

Upon completing the PAL task, participants were asked to fill out an additional 17 demographic and health/disease risk factor questions. These questions included: marital status, handedness, race, ethnicity, number of daily prescription medications, a first-degree family history of dementia, and yes/no responses to the following: seizures, dizzy spells, loss of consciousness (more than 10 min), high blood pressure, smoking status, diabetes mellitus, heart disease, cancer, reported stroke, alcohol/drug abuse, brain disease, and memory problems). Next, participants were shown their results and provided different comparisons to other test takers based on the average scores across all participants’ sex, age, and education demographics. On this same page of the site, participants were given the option to be recontacted for future research (see Supplementary Table 5 for the list of MindCrowd questions asked).

### Study participants UK Biobank: study design and aims

The UK Biobank is a long-term study and research resource in the United Kingdom (UK), which investigates links between genetic and environmental exposure to disease development. The UK Biobank’s stated goal is to “build a major resource that can support a diverse range of research intended to improve the prevention, diagnosis, and treatment of illness and the promotion of health throughout society.” The UK Biobank began in 2006. The study is currently following about 500,000 participants in the UK, enrolled at ages 40 to 69. Initial enrollment took place from 2006 to 2010. All participants are monitored for at least 30 years after recruitment and initial assessment (i.e., termed “instance 0” by the Biobank). Potential participants were invited to visit an assessment center, where they completed a questionnaire. Participants were next interviewed about lifestyle, medical history, and nutritional habits. Lastly, vital measurements, such as weight, height, and blood pressure, were measured. The UK Biobank aims to electronically record all health-related changes and events across the entire 30-year study. Notably, this task is aided by the UK’s integrated health system and corresponding electronic health record-keeping, an approach that is not yet possible in the USA.

### Study participants UK Biobank: data procurement

All UK Biobank data were derived from Application #43036, entitled “Exploring and Accommodating Heterogeneity in Large-Scale Genetic Analyses” as a “Collaborator Project.” The authors confirm that the UK Biobank obtained informed consent from each participant and complied with all relevant ethical regulations. Approval for this study was obtained from the Research Ethics Committee [11/NW/0382].

### Study participants UK Biobank: complex visual recognition reaction time (cvrRT) and educational attainment

Each participant’s cvrRT was based on 12 rounds of the card-game Snap. Participants were shown two cards at a time with a picture on them. Participants pressed a button on a table in front of them as quickly as possible if the images cards/matched. For each of the 12 rounds, the following data were collected: the pictures shown on the cards (Index of card A, Index of card B), the number of times the participant clicked the ‘snap’ button, and the latency to first click of the ‘snap’ button. This last record of “latency to click the button” was used as the UK Biobank’s criterion for regression analyses.

For Educational Attainment, the following conversions from UK Biobank (UKBb) answer codes (see http://biobank.ndph.ox.ac.uk/showcase/coding.cgi?id=100305) to MindCrowd (MC) values were made: (a) “UKBb **-7** None of the above” to “MC No high school diploma,” (b) “UKBb **2**A levels/AS levels or equivalent” to “MC High school diploma,” (c) “UKBb **3** O levels/GCSEs or equivalent” to “MC High school diploma,” (d) “UKBb **4** CSEs or equivalent” to “MC High school diploma,” (e) “UKBb **5** NVQ or HND or HNC or equivalent” to “MC Some college,” (f) “UKBb **6** Other professional qualifications (e.g., nursing and teaching)” to” MC Some college,” (g) “UKBb **1** College or University degree” to “MC College degree.” All UKBb participants selecting “**-3** Prefer not to answer” were removed from the final dataset before model selection and analysis. While we did our best to ensure a similar education measure across UKBb MindCrowd and the UK Biobank, we realize that there are fundamental differences between US and UK schools that we cannot control or eliminate. Table 6 lists the specific UK Biobank data fields from which we derived our factors.

**Table 6:** UK Biobank data fields used to derive factors.

Index #	Field description	Field ID	Count (*n*)	Type	Description	Column label	Used to generate
6	http://biobank.ndph.ox.ac.uk/showcase/field.cgi?id=31	31	502,524	Categorical (single)	Sex	Sex	Biological Sex
7	http://biobank.ndph.ox.ac.uk/showcase/field.cgi?id=34	34	502,524	Integer	Year of birth	Birth year	Age
264	http://biobank.ndph.ox.ac.uk/showcase/field.cgi?id=404	404	202,464	Integer	Duration to the first press of snap-button in each round	Snap-button time to first press (Instance 0)	Median cvrRT(8 out 12 Rounds)
395	http://biobank.ndph.ox.ac.uk/showcase/field.cgi?id=1707	1707	501,626	Categorical (single)	Handedness (chirality/laterality)	Handedness	Handedness
422	http://biobank.ndph.ox.ac.uk/showcase/field.cgi?id=2443	2443	501,593	Categorical (single)	Diabetes diagnosed by doctor	Diabetes mellitus	Diabetes mellitus
426	http://biobank.ndph.ox.ac.uk/showcase/field.cgi?id=2453	2453	501,593	Categorical (single)	Cancer diagnosed by doctor	Cancer	Cancer
482	http://biobank.ndph.ox.ac.uk/showcase/field.cgi?id=2966	2966	134,645	Integer	Age high blood pressure diagnosed	Age hypertension diagnosed	Hypertension Diag. via MD
616	http://biobank.ndph.ox.ac.uk/showcase/field.cgi?id=4056	4056	7,579	Integer	Age Reported Stroke diagnosed	Age reported stroke diagnosed	Reported Stroke Diag. via MD
2104	http://biobank.ndph.ox.ac.uk/showcase/field.cgi?id=20107	20107	487,790	Categorical (single)	Illnesses of father	(Multiple columns)	FHAD
2144	http://biobank.ndph.ox.ac.uk/showcase/field.cgi?id=20110	20110	492,928	Categorical (single)	Illnesses of mother	(Multiple columns)	FHAD
2188	http://biobank.ndph.ox.ac.uk/showcase/field.cgi?id=20111	20111	433,922	Categorical (single)	Illnesses of siblings	(Multiple columns)	FHAD
2325	http://biobank.ndph.ox.ac.uk/showcase/field.cgi?id=20116	20116	501633	Categorical (single)	Smoking status	Smoking status	Smoking Status
2356	http://biobank.ndph.ox.ac.uk/showcase/field.cgi?id=21022	21022	502,524	Integer	Age at recruitment	Age at recruitment	Age (verify)
120	http://biobank.ndph.ox.ac.uk/showcase/field.cgi?id=401	401	495,702	Categorical (single)	Index for card A in round	Needed to know if snap-button should have been pressed	Median cvrRT
168	http://biobank.ndph.ox.ac.uk/showcase/field.cgi?id=402	402	495,702	Categorical (single)	Index for card B in round	Needed to know if snap-button should have been pressed	Median cvrRT
1635	http://biobank.ndph.ox.ac.uk/showcase/field.cgi?id=6138	6138	497,883	Categorical (multiple)	Qualifications	Education	Educational attainment
2357	http://biobank.ndph.ox.ac.uk/showcase/field.cgi?id=21053	21053	174774	Categorical (single)	Degree bothered by dizziness in the last three months	Dizziness	Dizziness

UK Biobank’s “Index Number,” “Field Description,” “Field ID,” and sample size (*n*s) for data field used to generate factors evaluated via multiple linear regression. Data fields were chosen to match the questions posed in MindCrowd. If multiple instances (e.g., 0–4, timepoints) were available for data, only the first (instance 0) was used. For cvrRT and FHAD, multiple columns were compiled to generate the factor. For the UKBb MindCrowd and UK Biobank analyses, participants were removed if their responses to a demographic, medical, health, and lifestyle question did not match the other cohort.

### Data quality control

For the MindCrowd analysis, a final data set, including all qualifying participants up to 3–17–2020, was generated. See Supplementary Fig. 9 for a flowchart detailing the following filtering steps. This dataset removed participants: (a) with duplicate email addresses (only first entry kept), (b) who did not complete all three rounds of the PAL test, (c) whose primary language was not English, (d) who was not between 18–85 years old, (e) whose RT trials were above or below 1.5 × the interquartile range (IQR) and (f) whose median svRT was above or below 1.5 × the IQR range of all participants of the same age (Supplementary Fig. 10 details RT and IQR exclusion). Participants from either study were removed if they were missing any data (listwise deletion). Lastly, for the UKBb MindCrowd and UK Biobank analysis, participants were removed if their responses to a demographic, medical, health, and lifestyle question did not match the other study. For example, participants in the UK Biobank who responded to the “Race” question with “Prefer Not to Answer” were removed. “Prefer Not to Answer” was not a choice MindCrowd participants were given on the “Race” question. Removing these participants was done to align UKBb MindCrowd and UK Biobank cohorts as much as possible.

### Statistical methods

Statistical analyses were conducted using R^62,63^ (v4.0.3). For all analyses, multivariate linear regression was performed using the general linear model (LM) to model Median svRT or Median cvrRT (i.e., criterion or dependent variable) as a function of either 24 (MindCrowd) or 11 predictors (UK Biobank analysis). For the MindCrowd analysis, svRT was modeled as a function of PAL Performance and Age raised to the power of three (i.e., to fit and estimate nonlinear associations). Most figures were created using “ggplot2” bundled together as a part of the R package, “tidyverse^64^”. Continuous by continuous interactions (i.e., simple slopes) were estimated using the R packages “interactions^65^,” “sandwich^66^,” “jtools^67^”. Categorical by categorical interactions were estimated using the R package, “emmeans”^68^. Adjustments for multiple comparisons were evaluated using Tukey’s method via the “emmeans” package. Missing data were assessed via the “finalfit^69^”, “visdat^70^”, and “naniar^71^” R packages (see Supplementary Table 6 for a complete list of resources).

All measurements were taken from distinct samples. Model fit and violations of parametric assumptions were evaluated separately in each model. Here, we evaluated different residual plots, assessing normality, homoscedasticity, outliers, residual autocorrelation, and multicollinearity. The MindCrowd LM included all 22 demographic questions, health, medical, and lifestyle questions. These questions were: Age, Biological Sex, Race, Ethnicity, Educational Attainment, Marital Status, Handedness, Daily Medications, Seizures, Reported Dizziness, Loss of Consciousness, Reported Hypertension, Smoking Status, Heart Disease, Reported Stroke, Alcohol/Drug Abuse, Diabetes Mellitus, Cancer, a First-Degree Family History of Alzheimer’s disease, history of brain disease, whether the test was taken on a mobile device, and the version of the MindCrowd site used. Not surprisingly, the device used to take the RT test in MindCrowd was associated with RT performance. For the UK Biobank analyses, these 11 variables included: Age, Biological Sex, Diabetes mellitus, Handedness, Reported Stroke, Reported Hypertension, Smoking Status, Reported Dizziness, Educational Attainment, and a First-Degree Family History of Alzheimer’s Disease. Examination of each model’s variance inflation factors (VIF) revealed no unexpected factors with a VIF > 5 (i.e., considered “highly” colinear by convention, see Supplementary Table 7).

The MindCrowd analyses included Age and PAL Performance as first through third-degree non-orthogonal polynomials (i.e., cubic regression). This choice was based on empirical evaluations, using Bayesian information criterion (BIC) weights (i.e., Schwarz weights)^72^. We generated, ran, and recorded BICs across seven models (i.e., base and first-degree through sixth-degree-[nonorthogonal] polynomial). BIC weights were calculated from raw BIC values using the “qPCR^73^” (v1.4–1) R for each model. The third-degree polynomial model reported the largest BIC weight, and it was 1.46E + 270 times more likely to occur than the base (no polynomial) model (BIC_HighW_ 9.99E − 01/BIC_LowW_6.82E − 271 = 1.46E + 270)^72^. It is worth noting that a prior study examining both complex and simple RT also included age as a third-degree polynomial. Other similarities included: a relatively large *n* = 7000, both sexes, an 18–94-year-old age range, and several RT findings^72^.

For the MindCrowd, UKBb MindCrowd, and UK Biobank cohorts, we used the R package “glmulti” (v1.0.8)^35^ to define our GLM models. *“*glmulti*”* uses full information criterion model selection vs. shrinkage regression methods (e.g., LASSO or LAR)^35^. We used *“*glmulti*”* to avoid the pitfalls of stepwise selection methods or unintentional biased introduced via manual or *p*-value-based model selection. We had *“*glmulti*”* define the “best” (i.e., lowest BIC) MindCrowd and UK Biobank models separately using its *“*genetic*”* algorithm method with *“*marginality*”* set to True. We chose BIC as opposed to other information criterion methods because BIC punishes for model complexity. Two rounds of model selection were run to find pairwise interactions due to package limitations (i.e., millions of potential models). For round 1, the optimal model contained only the main effects when all 22 factors were included. In round 2, the only factors selected in the optimal main effects model were then included to select an optimal model, including two-way interactions.

### Reporting summary

Further information on research design is available in the Nature Research Reporting Summary linked to this article.

## Supplementary information

Supplementary Information

Reporting Summary

## Data Availability

All programs, software, and other materials described herein are publicly available. These data from MindCrowd that supports each analysis, figure, and table are freely available at Dryad (10.5061/dryad.j6q573ndg). UK Biobank data are available for researchers who meet the criteria and gain approval to access the research database. Access requests are reviewed, and authorizations are granted once ethical, and other UK Biobank criteria are met. Visit https://www.ukbiobank.ac.uk/enable-your-research/apply-for-access for information on how to gain access, as well as common inquiries and contact information. The complete code for the UK Biobank analyses is available at the Dryad link above, filename “mcsvrt_notebook_04132021.Rmd.” Aggregate MindCrowd and UK Biobank data analyzed in this study are available from the corresponding author on reasonable request.

## Code availability

The code supporting each analysis, figure, table, or other material is publicly available. R code used for all analyses and figure generation was included as “Source Data” R Markdown notebook. The R code includes each model selected and the complete list of parameters used. The directory containing these files is included in this study’s data repository (see Data availability for more information). The R code or scripts supporting this publication are publicly available and included as “mcsvrt_notebook_04132021.Rmd” R Markdown notebook. The directory containing these files is publicly available and included in this study’s data repository (https://doi.org/10.5061/dryad.j6q573ndg). Please see Supplementary Table 6 for a complete list of sourced software and resources (e.g., R and R packages) and their corresponding version.
