# Supplementary material for: Two separate, large cohorts reveal potential modifiers of age-associated variation in visual reaction time performance
Source: NPJ Aging Mech Dis. 2021 Jul 1;7:14. doi: 10.1038/s41514-021-00067-6 (PMC8249619; doi:10.1038/s41514-021-00067-6)
Supplement: Supplementary file 1 — Supplementary Information [file 41514_2021_67_MOESM1_ESM.pdf]

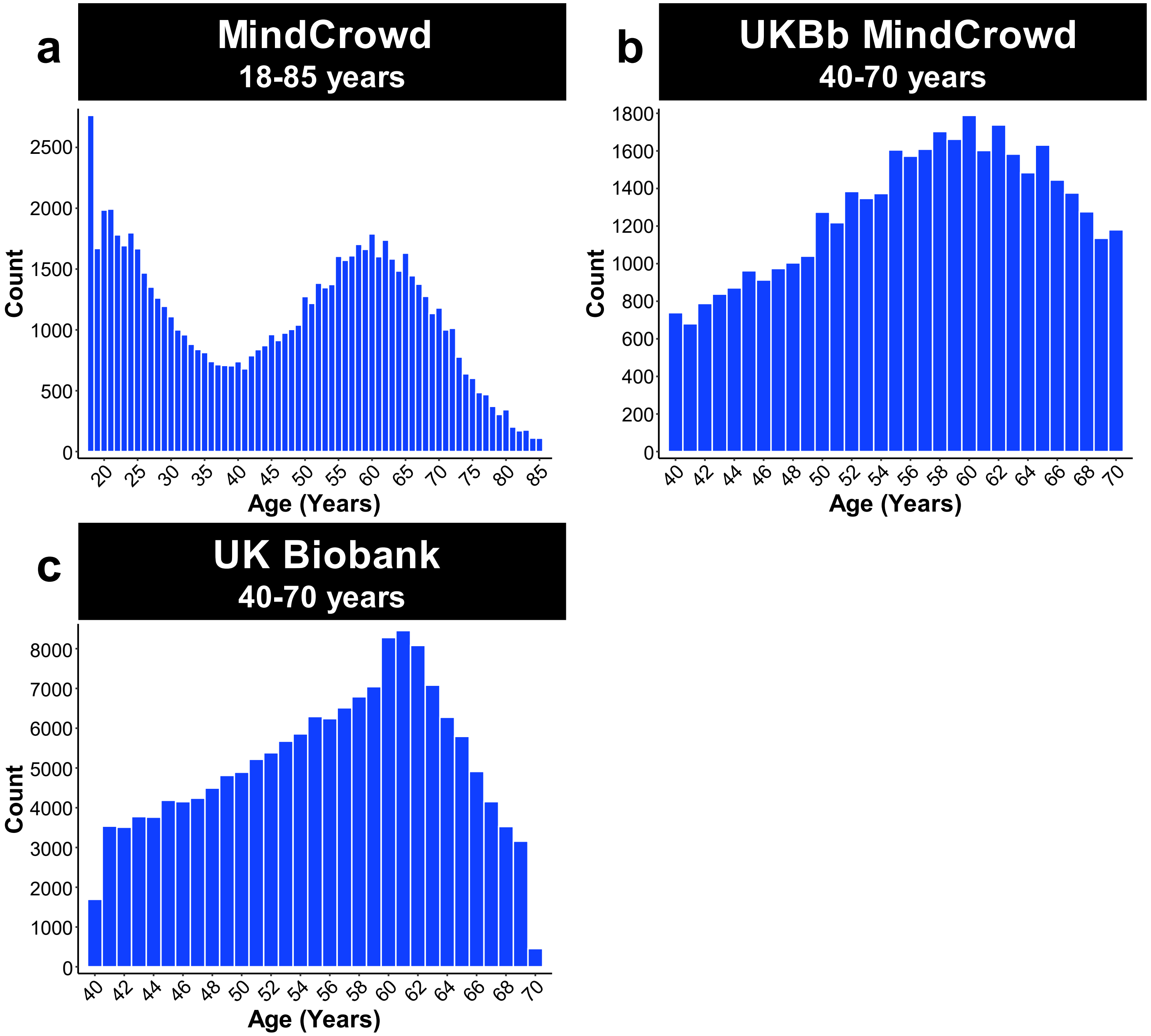

Supplementary Figure 1. Distribution of ages spanning MindCrowd, UKBb MindCrowd, and the UK Biobank. Histogram of age across (a) MindCrowd (18-85 years, n = 73,464), (b) UKBb MindCrowd (40-70 years, n = 38,891), and (c) UK Biobank (40-70 years, n = 157,933) cohorts. Each bin is one year. These plots display the bimodal distribution of ages in the MindCrowd cohort and the similar and approximately normal distribution of the UKBb MindCrowd and UK Biobank cohorts.

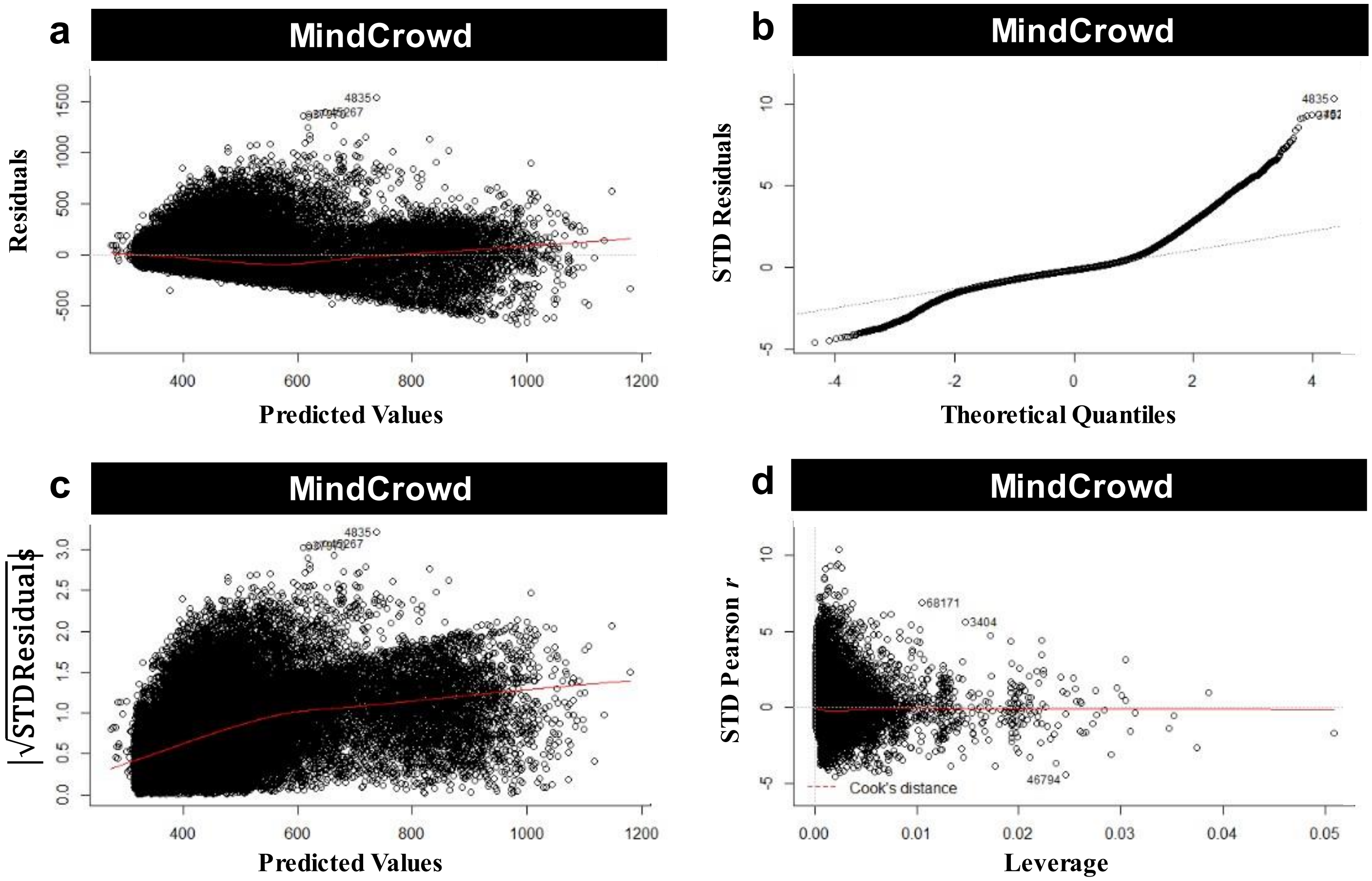

Supplementary Figure 2. Regression diagnostic plots of the general linear model (LM) for the MindCrowd analyses (*n* = 75,666). (a) A plot of the residuals versus fitted values. This plot suggests that the assumptions of linearity, equality of variances, and no outliers are met. (b) Q-Q plot displaying the quantiles of the data versus quantiles of a normal distribution. This plot suggests a violation of normality in residuals and thus the error terms; however, this violation should not cause major problems because of the large sample size. (c) A plot of the absolute value of the standardized (STD) residuals' square root versus the fitted values. Like plot A, this plot suggests variability in residuals does not change much over the range of the dependent variable. (d) A plot of the residuals versus leverage. This plot shows that there are no influential cases, as all cases fall within Cook's distance.

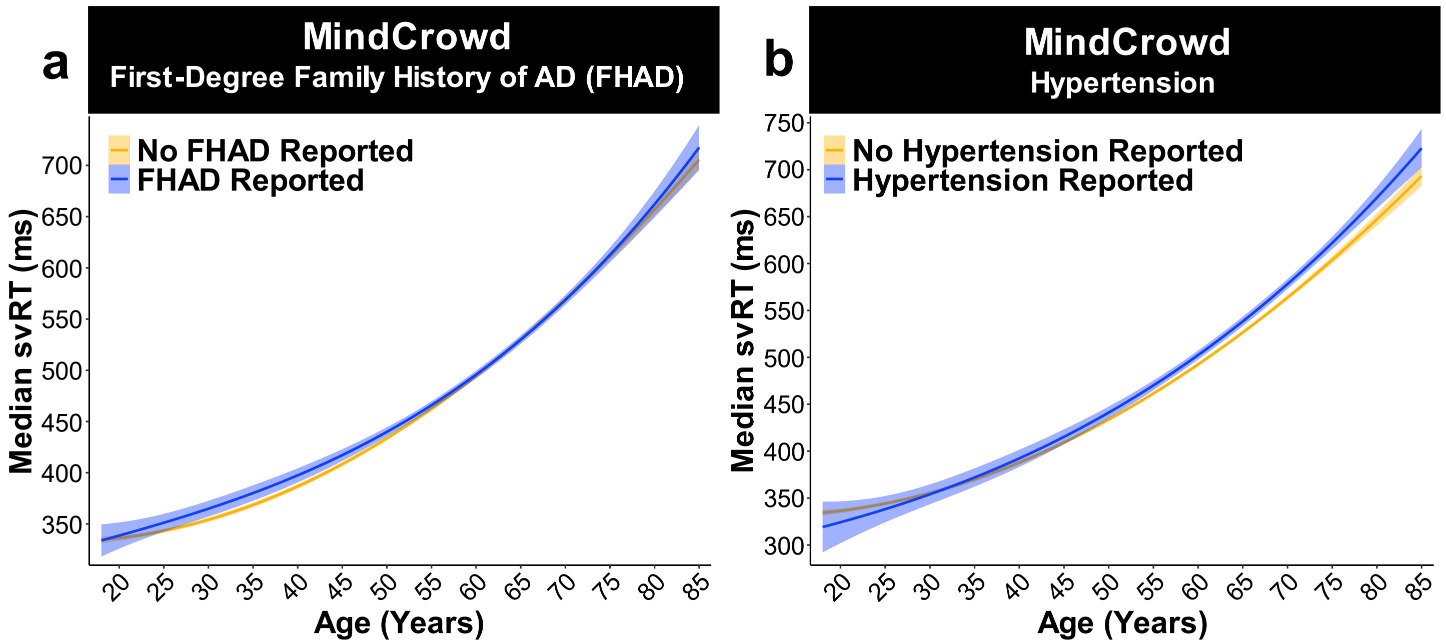

Supplementary Figure 3. MindCrowd analysis (ages 18-85): No relationship between a first-degree family history of Alzheimer's disease (FHAD) or Reported Hypertension and simple visual reaction time (svRT). (a) Linear model fits (line fill ± 95% *CI*) of the median svRT across Age with lines split by FHAD. There was no relationship between FHAD and svRT (β_{FHAD} = -0.40, p_{FHAD} = 0.75, FHAD Reported *n* = 17,847, No FHAD Reported *n* = 57,819). (b) Linear model fits (line fill ± 95% *CI*) of the median svRT across Age with lines split by Reported Hypertension. Hypertension was not related to svRT (β_{Hyper} = 1.08, p_{Hyper} = 0.52, Hypertension Reported *n* = 13,915, No Hypertension Reported *n* = 61,751).

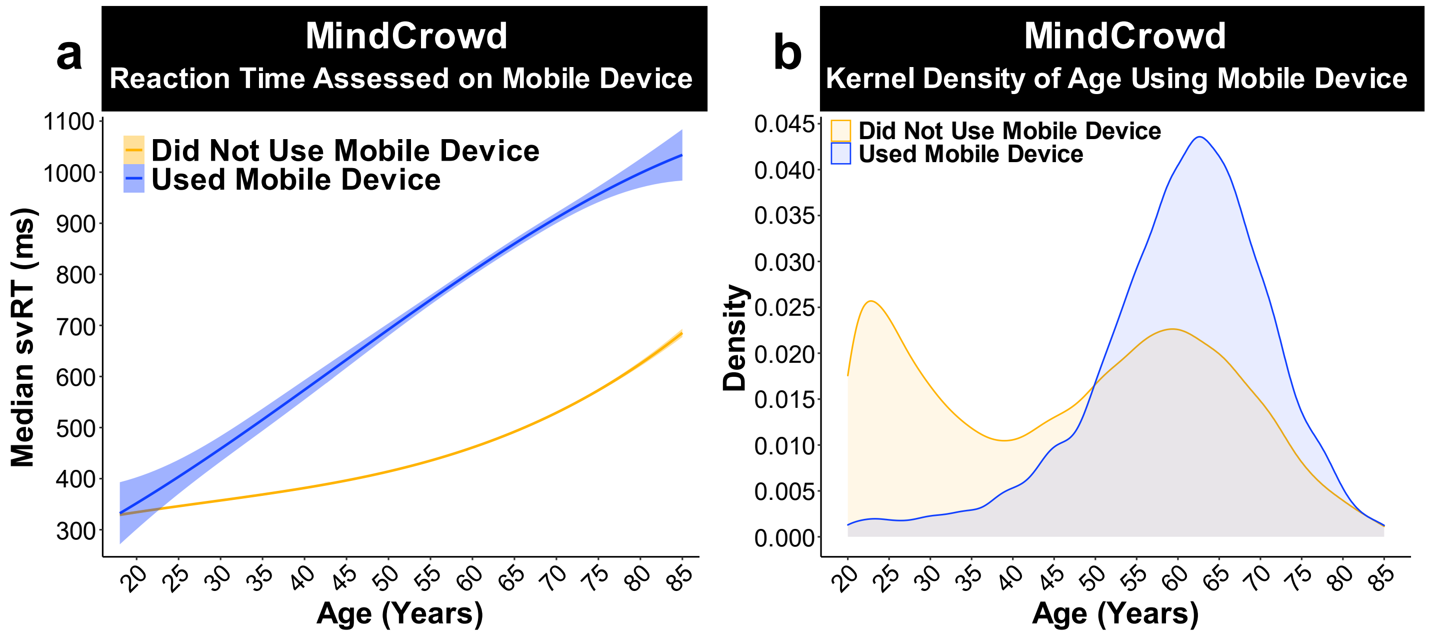

Supplementary Figure 4. MindCrowd analysis (ages 18-85): Lengthened simple visual reaction time (svRT) when using a mobile device. (a) linear model fits (line fill ± 95% *CI*) of the median svRT by Age^3 (curvilinear model). Touch screen devices were related to markedly longer svRT performance across age (β_{Device} = 322.30, $p_{Device} \leq$ 2e-16, Used Mobile Device *n* = 7,603 age *M* = 54.06 *SD* = 14.54, Did Not Use Mobile Device *n* = 76,775 age *M* = 45.54 *SD* = 18.43). (b) Kernel density plot of the age distribution of participants using a mobile device. Participants using a mobile device. Those using a mobile device (i.e., using a touchscreen, *n* = 7,603, age *M* = 54.06 *SD* = 14.54 years) had longer svRTs and were older ($\beta_{Age\sim Mobile}$ = 14.13, $p_{Age\sim Mobile}$ < 2e-16) than those who did not use a mobile device. The MindCrowd svRT task procedure was as follows. Participants were presented with a pink sphere that appeared at random intervals (between 1-10s) on the screen, and they were instructed to respond as quickly as possible after the sphere appeared by pressing the enter or return key on their keyboard. Once the participant responded, the sphere disappeared until the next trial. Each participant received a total of five trials. The sphere stayed on the screen until the participant responded. The dependent variable, the response time (milliseconds), was recorded from the sphere's appearance on the screen to the participant's key press or screen touch.

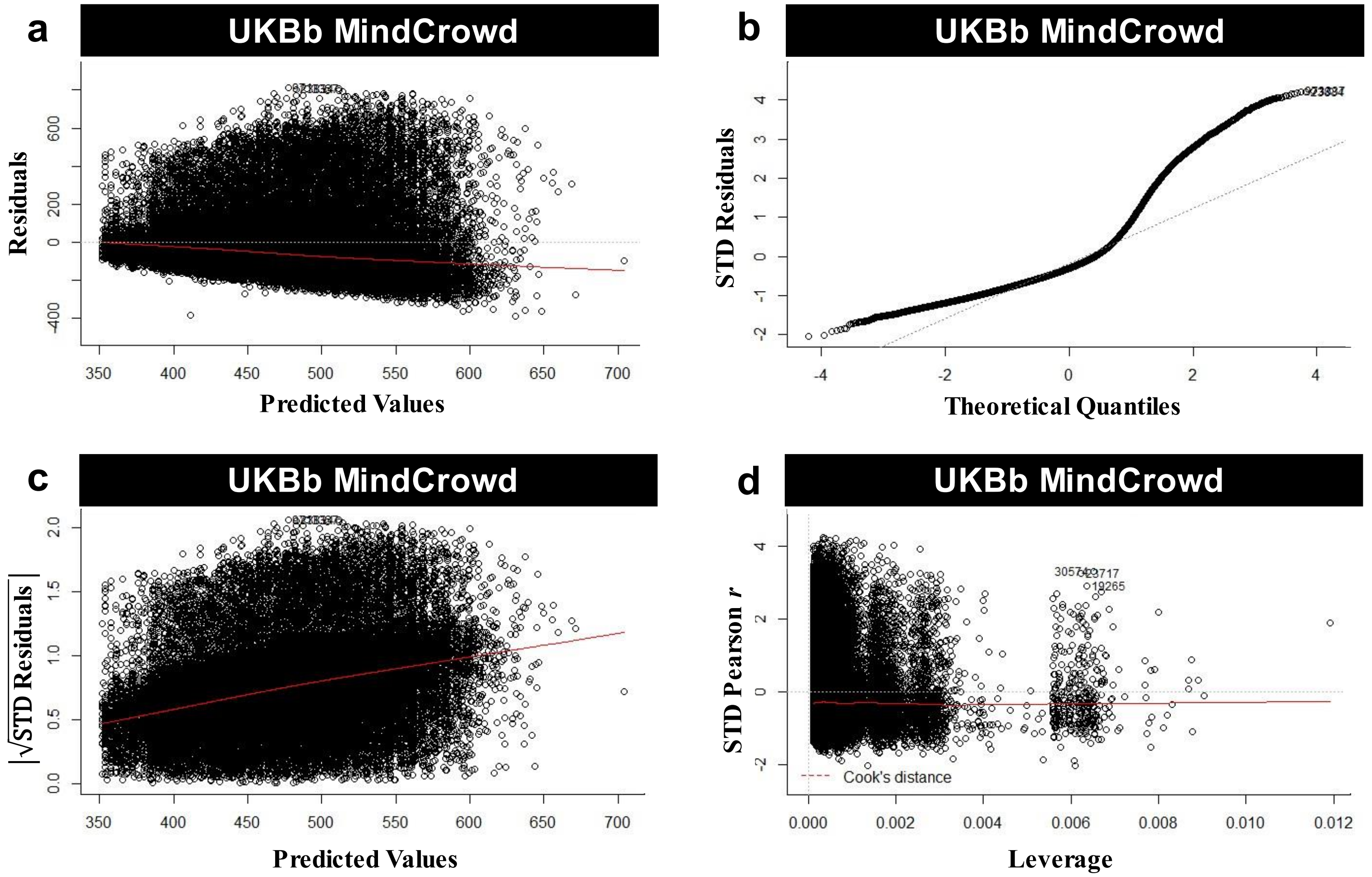

Supplementary Figure 5. Regression diagnostic plots of the general linear model (LM) for the UKBb MindCrowd analyses (*n* = 39,795). (a) A plot of the residuals versus fitted values. This plot suggests that the assumptions of linearity, equality of variances, and no outliers are met. (b) Q-Q plot displaying the quantiles of the data versus quantiles of a normal distribution. This plot suggests a violation of normality in residuals and thus the error terms; however, this violation should not cause major problems because of the large sample size. (c) A plot of the absolute value of the standardized (STD) residuals' square root versus the fitted values. Like plot A, this plot suggests variability in residuals does not change much over the range of the dependent variable. (d) A plot of the residuals versus leverage. This plot indicates that there are no influential cases, as all cases fall within Cook's distance.

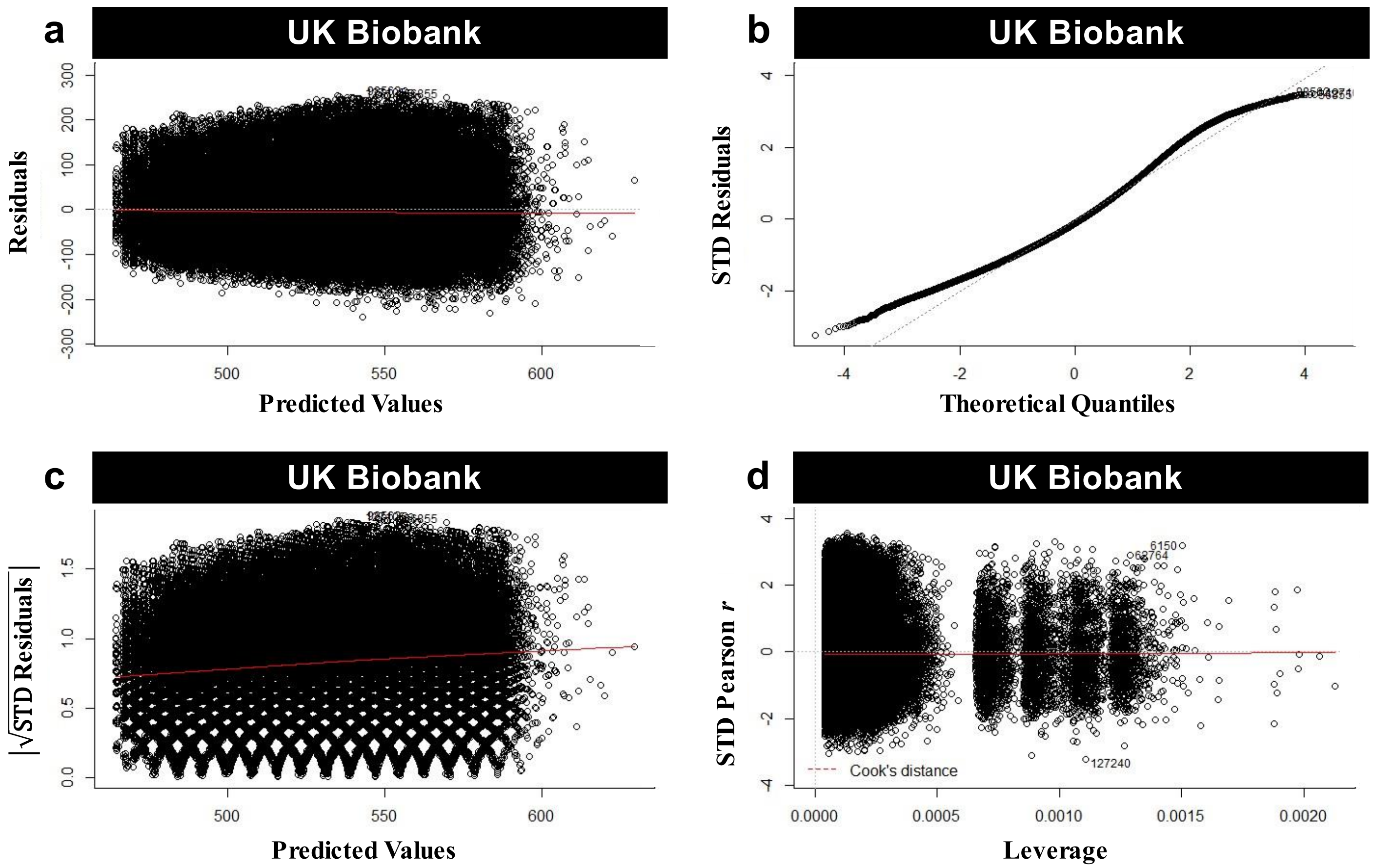

Supplementary Figure 6. Regression diagnostic plots of the general linear model (LM) for the UK Biobank analyses (*n* = 157,933). (a) A plot of the residuals versus fitted values. This plot suggests that the assumptions of linearity, equality of variances, and no outliers are met. (b) Q-Q plot displaying the quantiles of the data versus quantiles of a normal distribution. This plot suggests there is an approximately normal distribution of the residuals and, thus, the error terms. (c) A plot of the absolute value of the standardized (STD) residuals' square root versus the fitted values. This plot suggests that variability in residuals does not change much over the range of the dependent variable. (d) A plot of the residuals versus leverage. This plot shows that there are no influential cases, as all cases fall within Cook's distance.

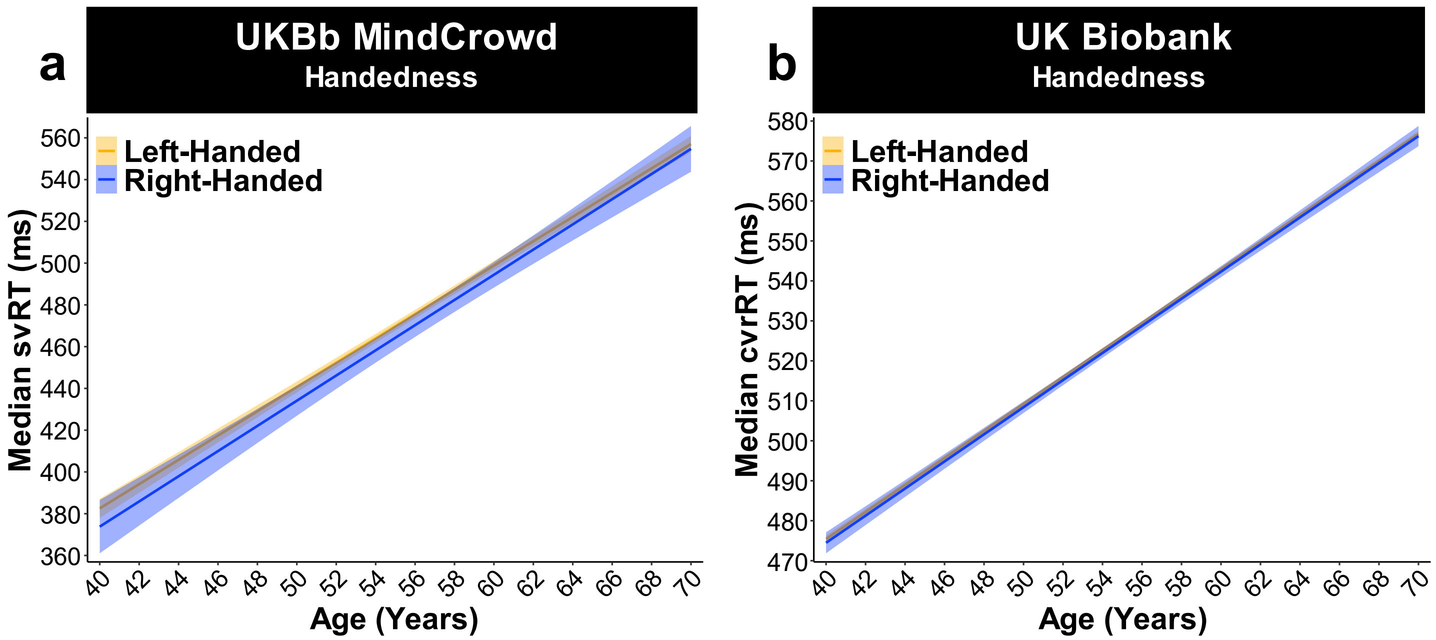

Supplementary Figure 7. UKBb MindCrowd and UK Biobank analysis (ages 40-70): Handedness was not associated with visual reaction time (RT). (a) Linear model fits (line fill ± 95% CI) of median simple visual RT (svRT). There was no relationship between reported handedness and svRT performance in the UKBb MindCrowd cohort. (b) Linear model fits (line fill ± 95% CI) of median complex, visual, recognition RT (cvrRT). For both the UKBb MindCrowd $\beta_{Handedness}$ = -2.58, $p_{Handedness}$ = 4.00E-01, Left-Handed *n* = 4,520, Right-Handed *n* = 35,034), and the UK Biobank ($\beta_{Handedness}$ = 0.58, $p_{Handedness}$ = 0.36, Left-Handed *n* = 15,287, Right-Handed *n* = 142,958), handedness was not related to cvrRT in the UK Biobank.

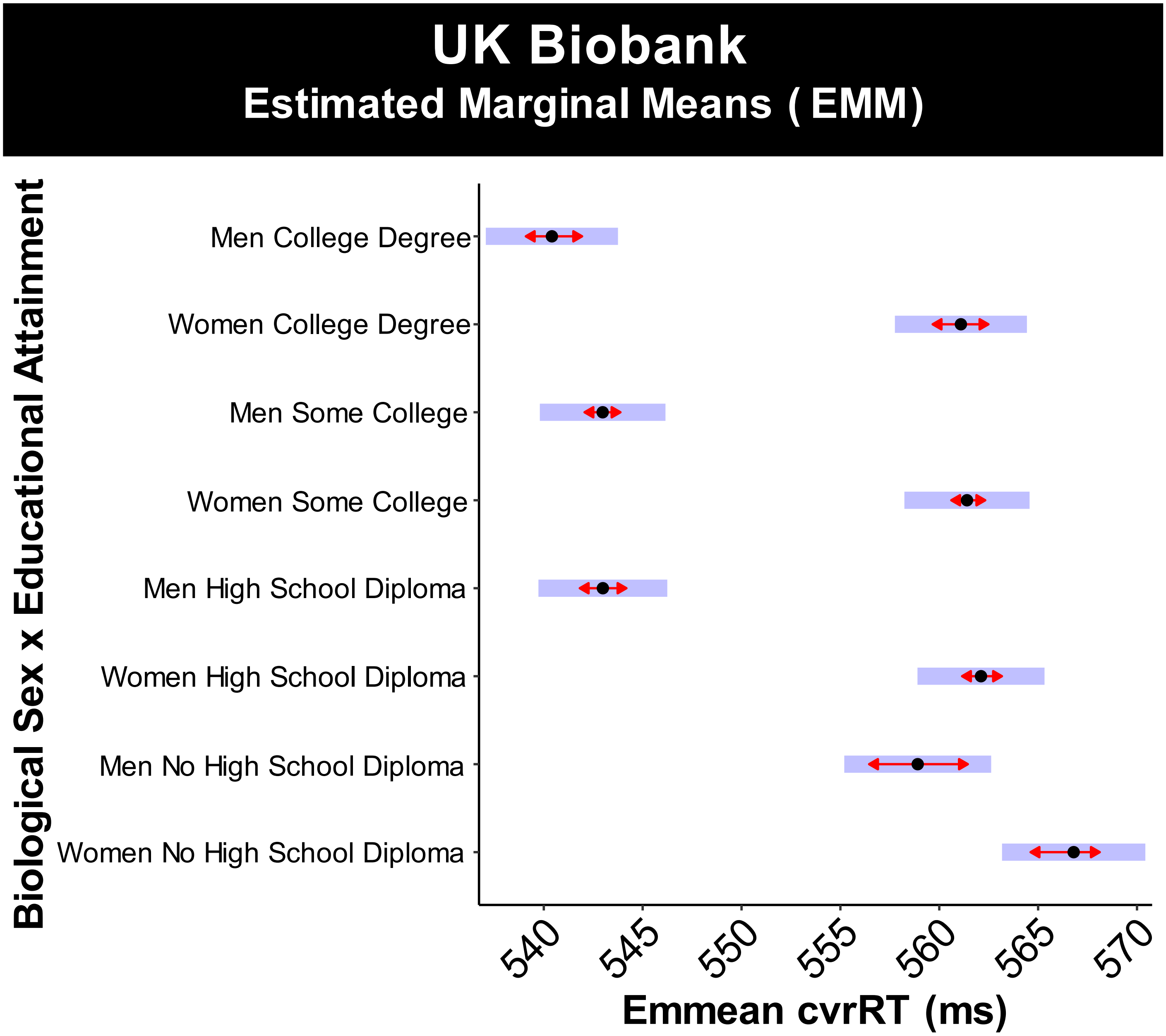

Supplementary Figure 8. Men with the lowest Educational Attainment show lengthened complex visual recognition reaction time (cvrRT). UK Biobank Biological Sex x Educational Attainment interaction (ages 40-70). Median cvrRT estimated marginal means (EMM) in the UK Biobank for the Biological Sex x Educational Attainment interaction. Purple/Blue rectangles display the range of the 95% confidence interval around the EMM, and the red arrows denote probable statistical significance if the arrows do not cross. Simple effects analysis via EMM revealed that men who did not graduate high school (EMM = 558.91 milliseconds), compared to men with more education (EMMs = 542.99,542.98, and 540.41 milliseconds), were associated with distinctly longer cvrRTs, more in line with the women's cvrRT performance (EMMs = 566.80, 562.11, 561.40, and 561.09 milliseconds). In addition, the difference in cvrRT between men at the two lowest education milestones (EMM = 558.91 vs. 542.99 milliseconds, βMen = 15.92, pMen = 2.00E-16, n = 4,922 and 18,505, 2.89%), was greater than those same two lowest milestones in women (EMM = 566.80 vs. 562.11 milliseconds, βWomen = 4.68, pWomen = 3.12E-04, n = 6,056 and 27,742 , 0.84%). See Figure 8 C-D for the Educational Attainment line graphs split by Biological Sex.

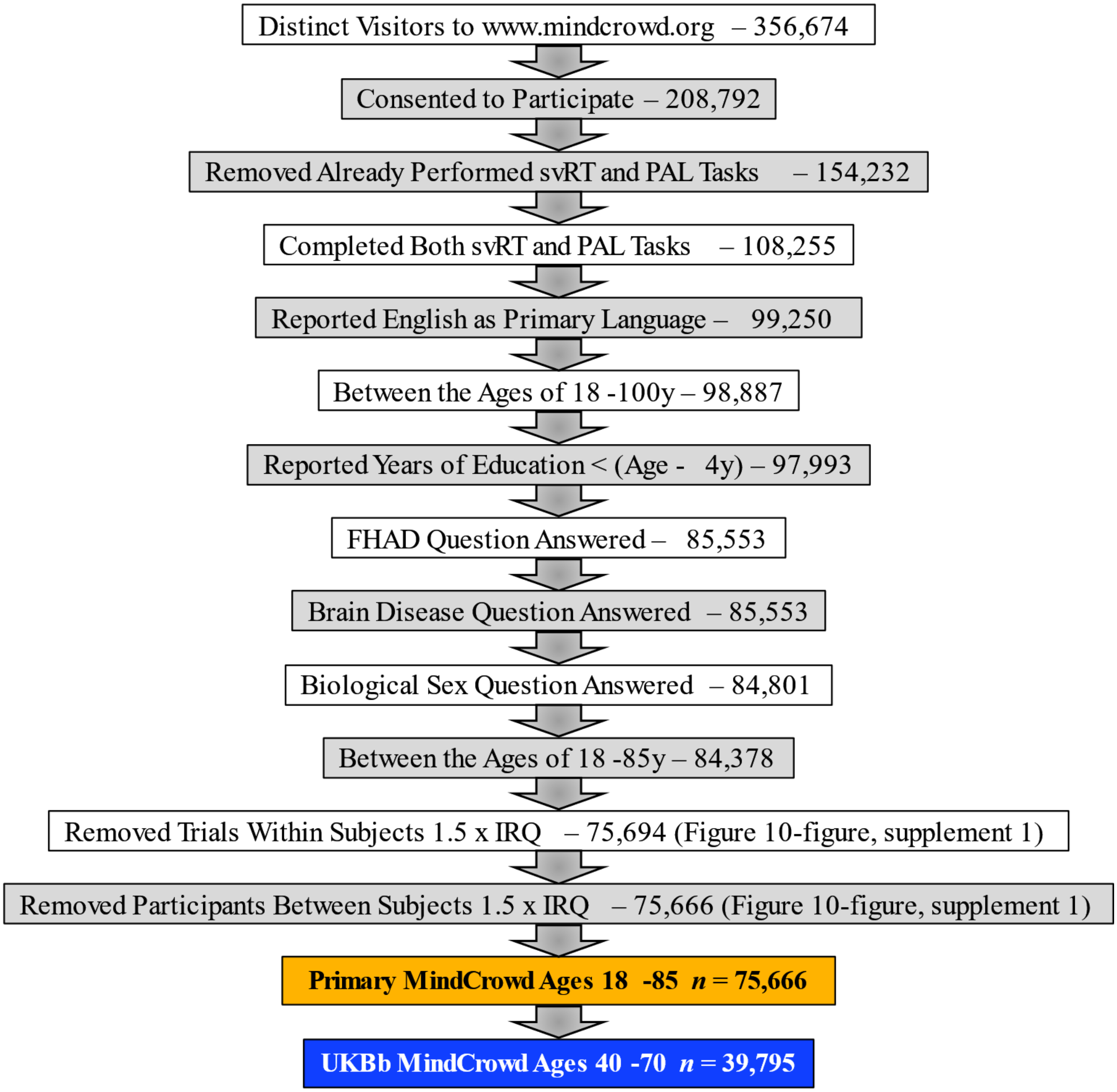

Supplementary Figure 9. Flowchart describing the data quality and control filtering steps, with associated change in *n* for each successive filtering step. This chart illustrates the primary filtering procedures used to ensure data quality for MindCrowd's online evaluation and self-report.

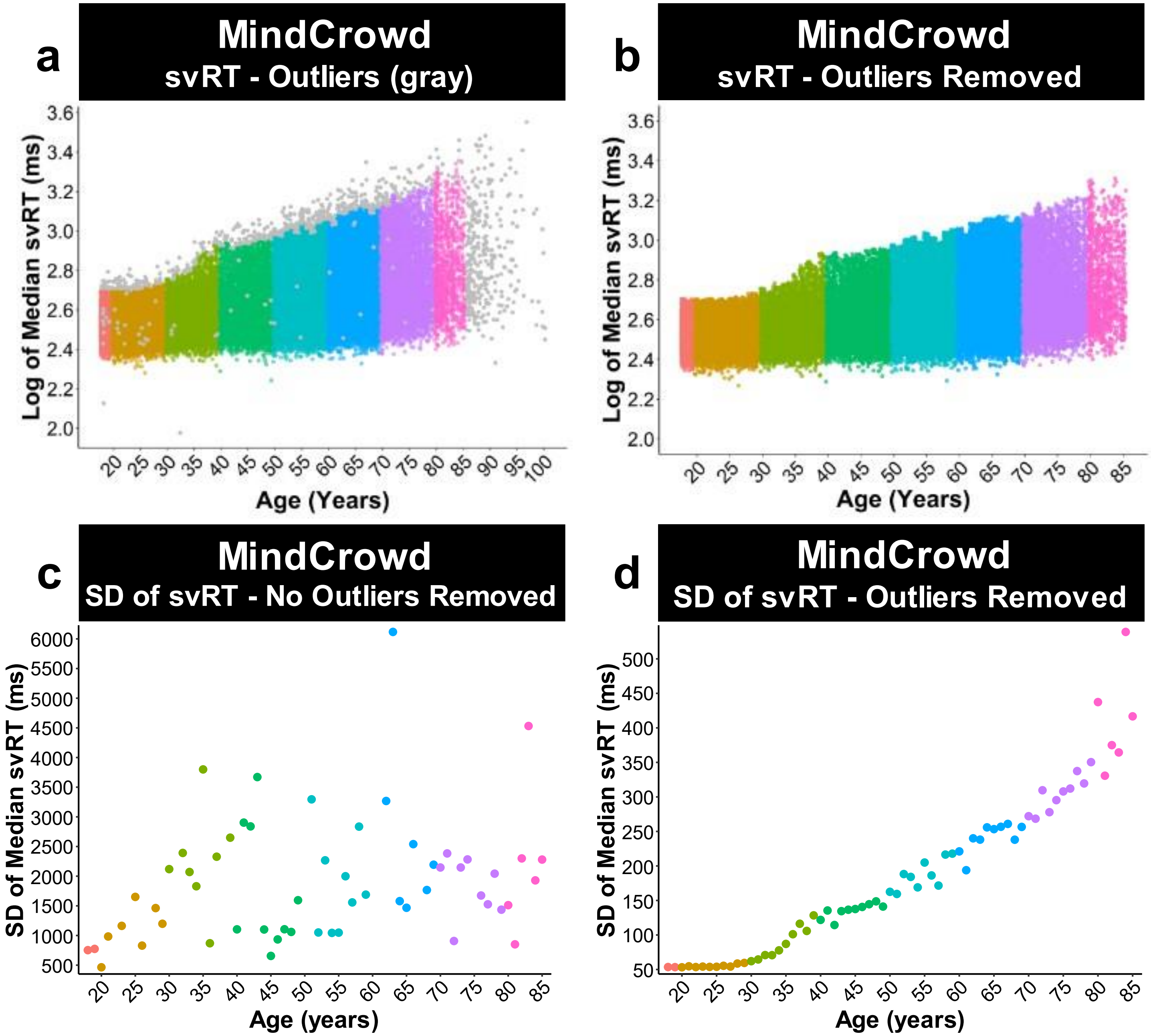

Supplementary Figure 10. Identification of simple visual reaction time (svRT) outliers and the effects of their removal. (a) The distribution of median svRT by Age in the entire MindCrowd cohort (n = 84,378). This graph displays outliers (svRT ± 1.5 x interquartile range [*IQR*] and age > 85y) shaded in gray. (b) A plot of the distribution of median svRT by Age once svRT outliers were removed (n = 75,666). (c) A plot of the standard deviation (*SD*) of median svRT by Age in the entire MindCrowd cohort with outliers (svRT ± 1.5 x *IQR*) present. (d) Graph of the *SD* of median svRT by Age in the MindCrowd cohort once outliers were removed. This graph illustrates the direct effect of outliers staying above or below 1.5 x IQR of these data's variation.

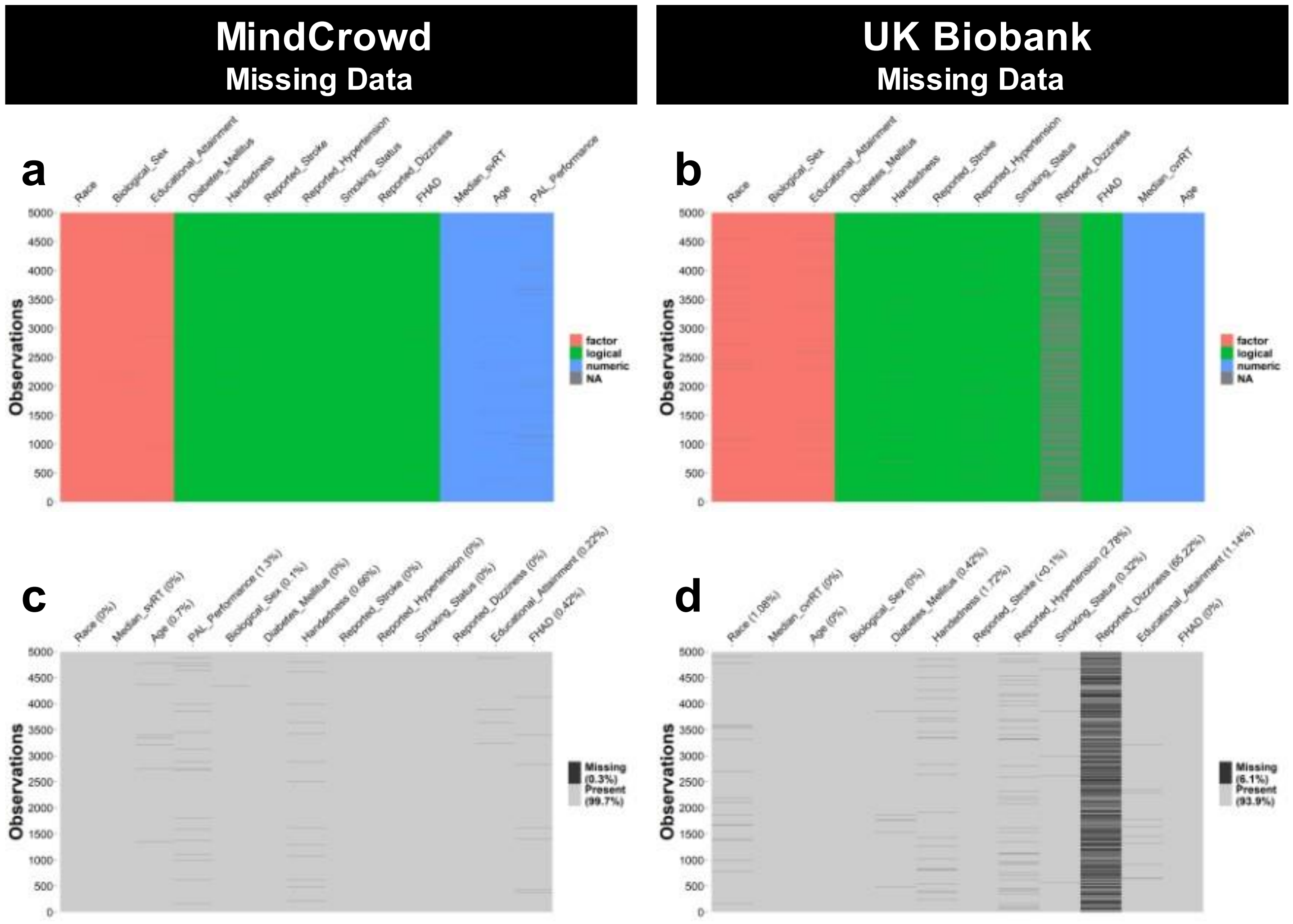

Supplementary Figure 11. Missing data patterns and percentages MindCrowd and the UK Biobank. The plot area of each graph displays 5000 random selected cases from a total *n* = 97,967 for (a-c) MindCrowd (ages 18-85) and an *n* = 201,139 for (b-d) the UK Biobank (ages 40-70). (a-b) Columns colored according to a factor's "Class." Gray (NA) denotes dropped or missing data. All cases with NAs were listwise deleted before analysis. (c-d) Display the overall study's "percent missing" and across each key factor. These percentages are derived from all the dataset cases, not the 5000 randomly selected to curtail graph size. The percent missing across both study cohorts and each key factor aside from Reported Dizziness in the UK Biobank was less than 5% (*M* = 0.50% *SD* = 0.74%). Reported Dizziness in the UK Biobank had 64.36% missing data. Thus, the scope of the interpretation of this factor's significant association with reaction time (RT) is restricted (see Opportunities and Limitations of Our Research in the Discussion). The mean percent missing for the one unique and 12 shared key factors in MindCrowd was less than 1% (*M* = 0.31%).). The mean percent missing for the 12 key shared factors in the UK Biobank was 7% (*M* = 7.04%). This average percent missing drops to less than 1% (*M* = 0.68%) when Reported Dizziness was excluded from the analysis.

Supplementary Table 1

MindCrowd Missing Data Information

Factor	Factor n	Missing n	Percent Missing
Age	96313	674	0.69
PAL Performance	95583	1404	1.45
Biological Sex	96916	71	0.07
Race	96987	0	0.00
Diabetes Mellitus	96987	0	0.00
Handedness	96436	551	0.57
Reported Stroke	96987	0	0.00
Reported Hypertension	96987	0	0.00
Smoking Status	96987	0	0.00
Reported Dizziness	96987	0	0.00
Educational Attainment	96691	296	0.31
FHAD	96589	398	0.41

Supplementary Table 1. Missing data numbers for MindCrowd. The Ten key factors in MindCrowd shared by the UK Biobank had relatively little missing data (merged reported and excluded n = 96,987, Missing Data Percentage M = 0.32% SD = 0.45%). Table acronym: A first-degree family history of Alzheimer's disease (FHAD).

Supplementary Table 2

UK Biobank Missing Data Information

Factor	Factor n	Missing n	Percent Missing
Age	201139	0	0
Biological Sex	201139	0	0
Race	198737	2402	1.2
Diabetes Mellitus	200511	628	0.31
Handedness	197525	3614	1.79
Reported Stroke	201040	99	< 0.00
Reported Hypertension	195867	5272	2.62
Smoking Status	200411	728	0.36
Reported Dizziness	71676	129463	64.36
Educational Attainment	199127	2012	1.00
FHAD	201139	0	0

Supplementary Table 2. Missing data numbers for the UK Biobank. Nine out of the ten key factors in the UK Biobank shared by MindCrowd had relatively little missing data (merged reported and excluded *n* = 201,139, *M* = 7.56%. *SD* = 17.65%). One potentially concerning factor in the UK Biobank was Reported Dizziness with 64.36% missing. Reported Dizziness had a significant association with cvrRT in our linear model. Interpretation of Reported Dizziness' association with complex visual recognition reaction time (cvrRT) should only be used for "hypothesis generation." Table acronym: A first-degree family history of Alzheimer's disease (FHAD).

Supplementary Table 3
MindCrowd: Odds Ratios (95% CIs) for Exclusion Due to Missing or Removed Data

Factor	Factor Level	Reported			Excluded			OR	95% CI
		n	%	Odds	n	%	Odds		
Age Decade	18-20	289	10.38	0.62	142	3.99	0.24	0.38	(0.48, 0.30)
	20-30	796	28.58	1.71	262	7.36	0.44	0.26	(0.31, 0.21)
	30-40	398	14.29	0.86	192	5.39	0.32	0.38	(0.46, 0.30)
	40-50	384	13.79	0.83	291	8.17	0.49	0.59	(0.72, 0.49)
	50-60[a]	465	16.70		597	16.77			
	60-70	343	12.32	0.74	587	16.49	0.98	1.33	(1.60, 1.11)
	70-80	100	3.59	0.22	323	9.07	0.54	2.52	(3.25, 1.95)
	80-90	10	0.36	0.02	72	2.02	0.12	5.61	(10.99, 2.86)
Biological Sex	Women	1,817	65.24	1.88	2272	63.82	1.87	0.99	(1.10, 0.90)
	Men	968	34.76		1217	34.19			
Race	Asian	1,570	56.37	3.51	229	6.43	0.08	0.02	(0.03, 0.02)
	Black	497	17.85	1.11	165	4.63	0.06	0.05	(0.06, 0.04)
	Mixed	271	9.73	0.61	300	8.43	0.10	0.17	(0.21, 0.14)
	White[a]	447	16.05		2866	80.51			
Educational Attainment	No High School Diploma[a]	137	4.92		507	14.24			
	High School Diploma	340	12.21	2.48	438	12.30	0.86	0.35	(0.44, 0.27)
	Some College	1,070	38.42	7.81	798	22.42	1.57	0.20	(0.25, 0.16)
	College Degree	1,238	44.45	9.04	1521	42.72	3.00	0.33	(0.41, 0.27)
Diabetes Mellitus	FALSE	2,574	92.42	0.08	3308	92.92	0.08	0.93	(1.12, 0.77)
	TRUE	211	7.58		252	7.08			
Handedness	FALSE	2,412	86.61	0.15	2670	75.00	0.13	0.87	(1.01, 0.74)
	TRUE	354	12.71		339	9.52			
Reported Stroke	FALSE	2,734	98.17	0.02	3495	98.17	0.02	1.00	(0.69, 0.69)
	TRUE	51	1.83		65	1.83			
Reported Hypertension	FALSE	2,189	78.60	0.27	2768	77.75	0.29	1.05	(1.19, 0.93)
	TRUE	596	21.40		792	22.25			
Smoking Status	FALSE	2,466	88.55	0.13	3235	90.87	0.10	0.78	(0.91, 0.66)
	TRUE	319	11.45		325	9.13			
Reported Dizziness	FALSE	2,534	90.99	0.10	3178	89.27	0.12	1.21	(1.43, 1.03)
	TRUE	251	9.01		382	10.73			
FHAD	FALSE	2,198	78.92	0.27	2385	66.99	0.33	1.22	(1.38, 1.08)
	TRUE	587	21.08		777	21.83			

Supplementary Table 3. Odds ratios (OR) with 95% CI for evaluation of selection bias within MindCrowd. Being excluded due to missing data (i.e., via listwise deletion) was less likely across most factor levels. Exceptions were participants over 60 years old were more likely excluded. In addition, participants reporting hypertension, dizziness, and a first-degree family history of Alzheimer's disease (FHAD) were more likely excluded. [a] denotes the factor level used as a reference for odds calculations.

Supplementary Table 4

The UK Biobank: Odds Ratios (95% CIs) for Exclusion to Missing or Removed Data

Factor	Factor Level	Reported			Excluded			OR	95% CI
		n	%	Odds	n	%	Odds		
Age Decade	40-50	38,167	24.12	0.64	30961	23.27	0.75	1.18	(1.20, 1.16)
	50-60[a]	59,904	37.85		41202	30.97			
	60-70	59,721	37.74	1.00	59952	45.06	1.46	1.46	(1.48, 1.44)
Biological Sex	Women	89,333	56.45	1.30	62484	46.96	0.89	0.68	(0.69, 0.67)
	Men	68,918	43.55		70571	53.04			
Race	Asian	847	0.54	0.01	777	0.58	0.01	1.31	(1.47, 1.19)
	Black	154,812	97.83	1.73	124003	93.20	1.98	1.15	(1.22, 1.13)
	Mixed	980	0.62	0.01	2465	1.85	0.02	3.14	(3.45, 2.91)
	White[a]	1,612	1.02		3408	2.56			
Educational Attainment	No High School Diploma[a]	10,979	6.94		28149	21.16			
	High School Diploma	46,248	29.22	4.21	37312	28.04	1.33	0.31	(0.32, 0.31)
	Some College	77,272	48.83	7.04	52680	39.59	1.87	0.27	(0.27, 0.26)
	College Degree	23,752	15.01	2.16	12902	9.70	0.46	0.21	(0.22, 0.21)
Diabetes Mellitus	FALSE	153,281	96.86	0.03	124202	93.35	0.07	2.04	(2.12, 2.01)
	TRUE	4,970	3.14		8225	6.18			
Handedness	FALSE	142,964	90.34	0.11	117170	88.06	0.10	0.98	(1.00, 0.97)
	TRUE	15,287	9.66		12271	9.22			
Reported Stroke	FALSE	157,013	99.22	0.01	130649	98.19	0.02	2.24	(2.40, 2.21)
	TRUE	1,238	0.78		2307	1.73			
Reported Hypertension	FALSE	125,655	79.40	0.26	93491	70.26	0.37	1.41	(1.44, 1.39)
	TRUE	32,596	20.60		34292	25.77			
Smoking Status	FALSE	63,953	40.41	1.47	52371	39.36	1.53	1.04	(1.05, 1.02)
	TRUE	94,298	59.59		79956	60.09			
Reported Dizziness	FALSE	116,040	73.33	0.36	2466	1.85	0.46	1.26	(1.35, 1.20)
	TRUE	42,211	26.67		1126	0.85			
FHAD	FALSE	138,509	87.52	0.14	118091	88.75	0.13	0.89	(0.91, 0.88)
	TRUE	19,742	12.48		14964	11.25			

Supplementary Table 4. Odds ratios (OR) with 95% CI for evaluation of selection bias within the UK Biobank. The odds of being excluded due to incomplete cases with missing data. The odds of being excluded due to missing data (i.e., listwise deletion) was more likely across over half of the key factor levels. Notable factor levels with a greater probability of being excluded were participants over 60 years old and those identified as Asian or Mixed race. In addition, participants reporting diabetes, stroke, hypertension, and dizziness also had a higher likelihood of being excluded. [a] denotes the factor level used as a reference for odds calculations.

Supplementary Table 5

MindCrowd: Participant Questions and How they were Code for Analysis

Question Number	Question Asked	Coded for Analysis
Pre-Test, Question 1	Sex:	Male, Female
Pre-Test, Question 2	Age:	Numeric Free Response
Pre-Test, Question 3	Primary Language:	1-54 (the number assigned to one of 54 major languages)
Pre-Test, Question 4	Education:	No High School Diploma<br>High School Diploma<br>Some College<br>College Degree
Pre-Test, Question 5	Country:	ISO 3166 international country code (e.g., TZ, UA, UG, US)
Post-Test, Question 1	What is your marital status?	Single, Married, Widowed, Unreported
Post-Test, Question 2	Are you left or right-handed?	True, False
Post-Test, Question 3	What is your Race?	American Indian or Alaska Native, Asian, Black/African American, Native Hawaiian/Pacific Islander, White, Mixed
Post-Test, Question 4	Are you of Hispanic, Latino, or Spanish origin?	True, False
Post-Test, Question 5	How many prescription medications do you take on a daily basis?	0, 1, 2, 3, 4
Post-Test, Question 6	Please check all the following that apply to you.	
6a	Loss of consciousness (> 10 minutes)	True, False
6b	Seizures	True, False
6c	Dizzy Spells	True, False
6d	High Blood Pressure	True, False
6e	Smoking Status	True, False
6f	Diabetes	True, False
6g	Heart Disease	True, False
6h	Cancer	True, False
6i	Reported Stroke	True, False
6j	Alcohol/Drug Abuse	True, False
6k	Brain Disease and/or Memory Problems	True, False
Post-Test, Question 7	Have you, a sibling, or one of your parents been diagnosed with Alzheimer’s disease?	True, False, Not Recorded

Supplementary Table 5. MindCrowd: Health, medical, lifestyle, and demographic questions. Table displaying questions participants were asked on the MindCrowd website. Other information for each question includes if it was asked per or post-testing, its number, and how it was coded for statistical analysis.

Supplementary Table 6

Key Resources

Resource Type	Designation	Source or Reference	Identifiers
Software, algorithm	R	The R Foundation	Version 4.0.3 RRID: SCR_001905
Software, algorithm	R package, tidyverse	Comprehensive R Archive Network (CRAN)	Version 1.3.0 RRID: SCR_014601: for ggplot2
Software, algorithm	R package, glmulti	Comprehensive R Archive Network (CRAN)	Version 1.0.7.1
Software, algorithm	R package, interactions	Comprehensive R Archive Network (CRAN)	Version 1.1.3
Software, algorithm	R package, sandwich	Comprehensive R Archive Network (CRAN)	Version 2.5-1
Software, algorithm	R package, jtools	Comprehensive R Archive Network (CRAN)	Version 2.0.5
Software, algorithm	R package, emmeans	Comprehensive R Archive Network (CRAN)	Version 1.4.6
Software, algorithm	RStudio	RStudio, PBC	Version 1.3.1093 RRID:SCR_000432
Software, algorithm	R package, finalfit	Comprehensive R Archive Network (CRAN)	Version 1.0.2
Software, algorithm	R package, visdat	Comprehensive R Archive Network (CRAN)	Version 0.5.3
Software, algorithm	R package, naniar	Comprehensive R Archive Network (CRAN)	Version 0.6.0
Software, algorithm	R package, qPCR	Comprehensive R Archive Network (CRAN)	Version 1.4-1

Supplementary Table 6. Key resources. Table displaying software and the version used in this study.

Supplementary Table 7

MindCrowd, UKBb MindCrowd, and the UK Biobank Variance Inflation Factors (VIF)

Factor	df	MindCrowd		UKBb MindCrowd		UK Biobank	
		VIF	VIF$^{(1/(2*Df))}$	VIF	VIF$^{(1/(2*df))}$	VIF	VIF$^{(1/(2*df))}$
Age^{1}	1	1076.88	32.82	1.49	1.22	1.95	1.40
Age^{2}	1	3491.06	59.09				
Age^{3}	1	961.20	31.00				
PAL Performance^{1}	1	159.95	12.65				
PAL Performance^{2}	1	751.04	27.41				
PAL Performance^{3}	1	275.83	16.61				
Biological Sex	1	7.81	2.79	106.20	10.31	78.48	8.86
Educational Attainment	3	231.58	2.48	2.45	1.16	6.06	1.35
Handedness	1	1.00	1.00	1.00	1.00	1.00	1.00
Daily Medications Taken	4	1.96	1.09				
Reported Dizziness	1	1.06	1.03	1.01	1.01	1.01	1.01
Smoking Status	1	7.60	2.76	1.03	1.02	1.03	1.01
Diabetes Mellitus	1	1.16	1.08	1.06	1.03	1.03	1.02
Reported Stroke	1	23.94	4.89	1.01	1.01	1.01	1.00
Reported Hypertension	1	1.37	1.17	1.10	1.05	1.08	1.04
FHAD	1	1.13	1.06	1.02	1.01	1.02	1.01
Age x PAL Performance	1	11.97	3.46				
Age x Smoking Status	1	7.40	2.72				
Age x Reported Stroke	1	23.95	4.89				
Age x Biological Sex	1	7.46	2.73	45.48	6.74	57.39	7.58
Age x Educational Attainment	3	2100.05	3.58	104.10	2.17	50.24	1.92

Supplementary Table 7. Summary of the variance inflation factors (VIF) across the three cohorts. These data suggest that the VIF for most factors was below conventional “high” multicollinearity levels (i.e., 5). The factors with VIF > 5 were expected to show higher values (e.g., polynomials, interaction terms, and dummy variables > three levels) and thus should not have meaningfully impacted model results.
