# Supplementary material for: Two separate, large cohorts reveal potential modifiers of age-associated variation in visual reaction time performance
Source: NPJ Aging Mech Dis. 2021 Jul 1;7:14. doi: 10.1038/s41514-021-00067-6 (PMC8249619; doi:10.1038/s41514-021-00067-6)
Supplement: Supplementary file 2 — Reporting Summary [file 41514_2021_67_MOESM2_ESM.pdf]

nature research

Corresponding author(s): Matt Huentelman, PhD

Last updated by author(s): 4/19/2021

Reporting Summary

Nature Research wishes to improve the reproducibility of the work that we publish. This form provides structure for consistency and transparency in reporting. For further information on Nature Research policies, see our Editorial Policies and the Editorial Policy Checklist.

Statistics

For all statistical analyses, confirm that the following items are present in the figure legend, table legend, main text, or Methods section.

n/a	Confirmed	
☐	☒	The exact sample size (*n*) for each experimental group/condition, given as a discrete number and unit of measurement
☐	☒	A statement on whether measurements were taken from distinct samples or whether the same sample was measured repeatedly
☐	☒	The statistical test(s) used AND whether they are one- or two-sided<br>*Only common tests should be described solely by name; describe more complex techniques in the Methods section.*
☐	☒	A description of all covariates tested
☐	☒	A description of any assumptions or corrections, such as tests of normality and adjustment for multiple comparisons
☐	☒	A full description of the statistical parameters including central tendency (e.g. means) or other basic estimates (e.g. regression coefficient) AND variation (e.g. standard deviation) or associated estimates of uncertainty (e.g. confidence intervals)
☐	☒	For null hypothesis testing, the test statistic (e.g. *F*, *t*, *r*) with confidence intervals, effect sizes, degrees of freedom and *P* value noted<br>*Give P values as exact values whenever suitable.*
☒	☐	For Bayesian analysis, information on the choice of priors and Markov chain Monte Carlo settings
☒	☐	For hierarchical and complex designs, identification of the appropriate level for tests and full reporting of outcomes
☒	☐	Estimates of effect sizes (e.g. Cohen's *d*, Pearson's *r*), indicating how they were calculated

Our web collection on statistics for biologists contains articles on many of the points above.

Software and code

Policy information about availability of computer code

Data collection	Ruby, Rails Framework, and PostgreSQL
Data analysis	R (v4.0.3)

For manuscripts utilizing custom algorithms or software that are central to the research but not yet described in published literature, software must be made available to editors and reviewers. We strongly encourage code deposition in a community repository (e.g. GitHub). See the Nature Research guidelines for submitting code & software for further information.

Data

Policy information about availability of data

All manuscripts must include a data availability statement. This statement should provide the following information, where applicable:

- Accession codes, unique identifiers, or web links for publicly available datasets
- A list of figures that have associated raw data
- A description of any restrictions on data availability

All programs, software, and other materials described herein are publicly available. These data from MindCrowd that supports each analysis, figure, and table is freely available at Dryad (https://doi.org/10.5061/dryad.j6q573ndg). UK Biobank data are available for researchers who meet the criteria and gain approval to access the research database. Access requests are reviewed and authorizations granted once ethical, and other UK Biobank criteria are met. Visit https://www.ukbiobank.ac.uk/enable-your-research/apply-for-access for information on how to gain access as well as common inquiries and contact information. The complete code for the UK Biobank analyses is available at https://doi.org/10.5061/dryad.j6q573ndg, filename “mcsvrt_notebook_04132021.Rmd.” Aggregate MindCrowd and UK Biobank data analyzed in this study are available from the corresponding author on reasonable request. The required statements regarding data and code availability are present in the main body of the manuscript.

Field-specific reporting

Please select the one below that is the best fit for your research. If you are not sure, read the appropriate sections before making your selection.

☐ Life sciences ☒ Behavioural & social sciences ☐ Ecological, evolutionary & environmental sciences

For a reference copy of the document with all sections, see nature.com/documents/nr-reporting-summary-flat.pdf

Behavioural & social sciences study design

All studies must disclose on these points even when the disclosure is negative.

Study description

A Quantitative, observational, and cross-sectional from two large separate (i.e., USA and UK) study cohorts

Research sample

Cohort	Descriptive or Factor Level	n	%
1. MindCrowd 18-85yr:	Age M = 46.54 SD = 18.40	75,666	100
UKBb MindCrowd 40-70yr:	Age M = 56.59 SD = 8.16	39,795	100
UK Biobank 40-70yr:	Age M = 55.81 SD = 7.72 1	158,249	100
2. MindCrowd 18-85yr: Biological Sex	Women	47,700	63.08
	Men	27,966	36.91
UKBb MindCrowd 40-70yr: Biological Sex	Women	29,640	74.51
	Men	10,155	25.49
UK Biobank 40-70yr: Biological Sex	Women	89,333	56.45
	Men	68,916	43.55
3. MindCrowd 18-85yr: Race	Native American	447	0.59
	Asian	3,511	4.64
	Black/African American	1,570	2.07
	Polynesian	271	0.36
	Mixed	497	0.66
	White	68,450	90.46
UKBb MindCrowd 40-70yr: Race	Asian	750	1.88
	Black/African American	740	1.86
	Mixed	185	0.46
	White	37,446	94.10
UK Biobank 40-70yr: Race	Asian	1,612	1.02
	Black	980	0.62
	Mixed	847	0.54
	White	154,810	97.83
4. MindCrowd 18-85yr: FHAD	TRUE	17,847	23.59
	FALSE	57,819	76.41
UKBB MindCrowd 40-70yr: FHAD	TRUE	13,784	34.59
	FALSE	26,047	65.41
UK Biobank 40-70yr: FHAD	TRUE	19,742	12.48
	FALSE	138,507	87.52
5. MindCrowd 18-85yr: Handedness	Left	8,449	11.17
	Right	66,903	88.42
UKBb MindCrowd 40-70yr: Handedness	Left	4,520	11.36
	Right	35,034	88.04
UK Biobank 40-70yr: Handedness	Left	15,287	9.66
	Right	142,962	90.34
6. MindCrowd 18-85yr:	No High School Diploma	1,881	2.49
Educational Attainment	High School Diploma	6,695	8.85
	Some College	22,950	30.33
	College Degree	44,140	58.34
UKBb MindCrowd 40-70yr:	No High School Diploma	605	1.52
Educational Attainment	High School Diploma	3,176	7.98
	Some College	10,978	27.99
	College Degree	24,875	62.51
UK Biobank 40-70yr:	No High School Diploma	10,978	6.94
Educational Attainment	High School Diploma	46,248	29.23
	Some College	77,271	48.83

College Degree	23,752	15.01

Sampling strategy

MindCrowd:
Web-based voluntary response sample. We chose sample sizes based on final participant numbers after filtering and quality control steps. The data were filtered in an attempt to control some of the limitations of this sampling procedure.

The UK Biobank:
With anywhere from 150,000 to over 500,000 participants, this is one of the most extensive cross-sectional studies in cognitive neuroscience; thus, the sample size is sufficient.

Data collection

MindCrowd:
Simple visual reaction time (svRT)
After consenting to the study and answering five demographic questions (i.e., age, biological sex, years of education, primary language, and country where they reside), participants were asked to complete a web-based svRT task. We chose svRT because it is a simple central and peripheral nervous system-dependent task influenced by intelligence and brain injury. Participants were presented with a pink sphere that appeared at random intervals (between 1-10 seconds) on the screen, and they were instructed to respond as quickly as possible after the sphere appeared by pressing the enter/return key on their keyboard. Once the participant responded, the sphere disappeared until the subsequent trial. Each participant received a total of five trials. The sphere stayed on the screen until the participant responded. The dependent variable, response time in milliseconds (msec), was recorded from the sphere's appearance on the screen to the participant's key press or screen touch.
Paired-associate learning (PAL)
Next, participants were presented with the PAL task. For this cognitive task, during the learning phase, participants were shown 12 word pairs, one word pair at a time (2s/word pair). During the recall phase, participants were given the first word of each pair and were asked to use their keyboard to type in (i.e., recall) the missing word. This learning-recall procedure was repeated for two more trials. Before beginning the task, each participant received one practice trial consisting of three word pairs not contained in the 12 used during the test. Word pairs were presented in different random orders during each learning and each recall phase. The same word pairs and order of presentation were used for all participants. The dependent variable/criterion was the total number of correct word pairs entered across the three trials (i.e., 12 x 3 = 36, a perfect score).
Demographic, medical, health, and lifestyle questions
Upon completing the PAL task, participants were asked to fill out an additional 17 demographic and health/disease risk factor questions. These questions included: marital status, handedness, race, ethnicity, number of daily prescription medications, a first-degree family history of dementia, and yes/no responses to the following: seizures, dizzy spells, loss of consciousness (more than 10 minutes), high blood pressure, smoking status, diabetes mellitus, heart disease, cancer, reported stroke, alcohol/drug abuse, brain disease, and memory problems). Next, participants were shown their results and provided different comparisons to other test takers based on the average scores across all participants' sex, age, and education demographics. On this same page of the site, participants were given the option to be recontacted for future research (see Supplementary Table 5 for the list of MindCrowd questions asked).

The UK Biobank:
Complex visual recognition reaction time (cvrRT) and educational attainment.
Each participant's cvrRT was based on 12 rounds of the card-game Snap. Participants were shown two cards at a time with a picture on them. Participants pressed a button on a table in front of them as quickly as possible if the images cards/matched. For each of the 12 rounds, the following data were collected: the pictures shown on the cards (Index of card A, Index of card B), the number of times the participant clicked the 'snap' button, and the latency to first click of the 'snap' button. This last record of "latency to click the button" was used as the UK Biobank's criterion for regression analyses.
For Educational Attainment, the following conversions from UK Biobank (UKBb) answer codes (see http://biobank.ndph.ox.ac.uk/showcase/coding.cgi?id=100305) to MindCrowd (MC) values were made: a) "UKBb -7 None of the above" to "MC No high school diploma," b) "UKBb 2 A levels/AS levels or equivalent" to "MC High school diploma," c) "UKBb 3 O levels/GCSEs or equivalent" to "MC High school diploma," d) "UKBb 4 CSEs or equivalent" to "MC High school diploma," e) "UKBb 5 NVQ or HND or HNC or equivalent" to "MC Some college," f) "UKBb 6 Other professional qualifications (e.g., nursing and teaching)" to" MC Some college," g) "UKBb 1 College or University degree" to "MC College degree." All UKBb participants selecting "-3 Prefer not to answer" were removed from the final dataset before model selection and analysis. While we did our best to ensure a similar education measure across UKBb MindCrowd and the UK Biobank, we realize that there are fundamental differences between US and UK schools that we cannot control or eliminate. Table 6 lists the specific UK Biobank data fields from which we derived our factors.

Timing

MindCrowd:
Start: January 2013. Stop: March 2020 (i.e., data freeze to write the manuscript as new participants visit, consent, and take the test daily).

The UK Biobank:
Began in 2006. The study is currently following about 500,000 participants in the UK, enrolled at ages 40 to 69. Initial enrollment took place from 2006 to 2010. All participants are monitored for at least 30 years after recruitment and initial assessment (i.e., termed "instance 0" by the Biobank).

Data exclusions

We developed an extensive and automated data filtering pipeline (see Data Quality Control and Supplementary Figures 9-10) to address these concerns and enhance validity and accuracy. These data (i.e., raw or filtered) were excluded before analysis (i.e., listwise deletion). Exclusion resulted in dropping 0.3 % and 6.1% of MindCrowd and UK Biobank participants, respectively.

MindCrowd:
A final data set, including all qualifying participants up to 3-17-2020, was generated. See Supplementary Figure 9 for a flowchart detailing the following filtering steps. This dataset removed participants: a) with duplicate email addresses (only first entry kept), b)

who did not complete all three rounds of the PAL test, c) whose primary language was not English, d) who was not between 18-85 years old, e) whose RT trials were above or below 1.5 x the interquartile range (IQR) and f) whose median svRT was above or below 1.5 x the IQR range of all participants of the same age (Supplementary Figure 10 details RT and IQR exclusion). Participants from either study were removed if they were missing any data (listwise deletion).

The UK Biobank:
Participants were removed if their responses to a demographic, medical, health, and lifestyle question did not match the other study. For example, participants in the UK Biobank who responded to the "Race" question with "Prefer Not to Answer" were removed. "Prefer Not to Answer" was not a choice MindCrowd participants were given on the "Race" question. Removing these participants was done to align UKBb MindCrowd and UK Biobank cohorts as much as possible.

Non-participation	Since both MindCrowd and evaluation of instance "0" from the UK Biobank were voluntary and cross sectional no participants dropped out or declined participation.
Randomization	As a result of MindCrowd and the UK Biobank being a voluntary cross-sectional study, participants were not allocated into groups. Self-identification of demographic, health, and lifestyle questions served as predictors/independent variables associating these factors to paired-associates learning performance. All 22 (MindCrowd) and 12 (UK Biobank) demographic, health, and lifestyle questions were included as covariates in our multiple regression (i.e., linear model) analyses.

Reporting for specific materials, systems and methods

We require information from authors about some types of materials, experimental systems and methods used in many studies. Here, indicate whether each material, system or method listed is relevant to your study. If you are not sure if a list item applies to your research, read the appropriate section before selecting a response.

Materials & experimental systems

n/a	Involved in the study
☒	☐ Antibodies
☒	☐ Eukaryotic cell lines
☒	☐ Palaeontology and archaeology
☒	☐ Animals and other organisms
☐	☒ Human research participants
☒	☐ Clinical data
☒	☐ Dual use research of concern

Methods

n/a	Involved in the study
☒	☐ ChIP-seq
☒	☐ Flow cytometry
☒	☐ MRI-based neuroimaging

Human research participants

Policy information about studies involving human research participants

Population characteristics	Please see "Research sample" above
Recruitment	MindCrowd:<br>In January 2013, we launched our internet-based study at www.mindcrowd.org. Website visitors 18 years or older were asked to consent to our study before any data collection via an electronic consent form. As of 3-17-2020, we have had 356,674 non-duplicate or distinct visitors to the website. Of these distinct visitors, over 194,542 (54%) consented to take part. The final data set had 75,666 (39% of consented individuals) participants who completed a simple visual reaction time (svRT) and paired-associate learning (PAL) tasks and answered 22 demographic, lifestyle, and health questions. The authors confirm they obtained informed consent from each participant and complied with all relevant ethical regulations.<br><br>The UK Biobank:<br>Potential participants were invited to visit an assessment center, where they completed a questionnaire. Participants were next interviewed about lifestyle, medical history, and nutritional habits. Lastly, vital measurements, such as weight, height, and blood pressure, were measured. The UK Biobank aims to electronically record all health-related changes and events across the entire 30-year study. Notably, this task is aided by the UK's integrated health system and corresponding electronic health record-keeping, an approach that is not possible in the USA.
Ethics oversight	MindCrowd:<br>The authors confirm they obtained informed consent from each participant and complied with all relevant ethical regulations. Approval for this study was obtained from the Western Institutional Review Board (WIRB study number 1129241).<br><br>The UK Biobank:<br>All UK Biobank data were derived from Application #43036, entitled "Exploring and Accommodating Heterogeneity in Large-Scale Genetic Analyses" as a "Collaborator Project." The authors confirm the UK Biobank obtained informed consent from each participant and complied with all relevant ethical regulations. Approval for this study was obtained from the Research Ethics Committee [11/NW/0382].

Note that full information on the approval of the study protocol must also be provided in the manuscript.
